# Supplementary material for: Comparison of insect and human cytochrome b561 proteins: Insights into candidate ferric reductases in insects
Source: PLoS One. 2023 Dec 1;18(12):e0291564. doi: 10.1371/journal.pone.0291564 (PMC10691727; doi:10.1371/journal.pone.0291564)
Supplement: S1 Fig — (PDF) [file pone.0291564.s001.pdf]

S1 Fig. Part 1

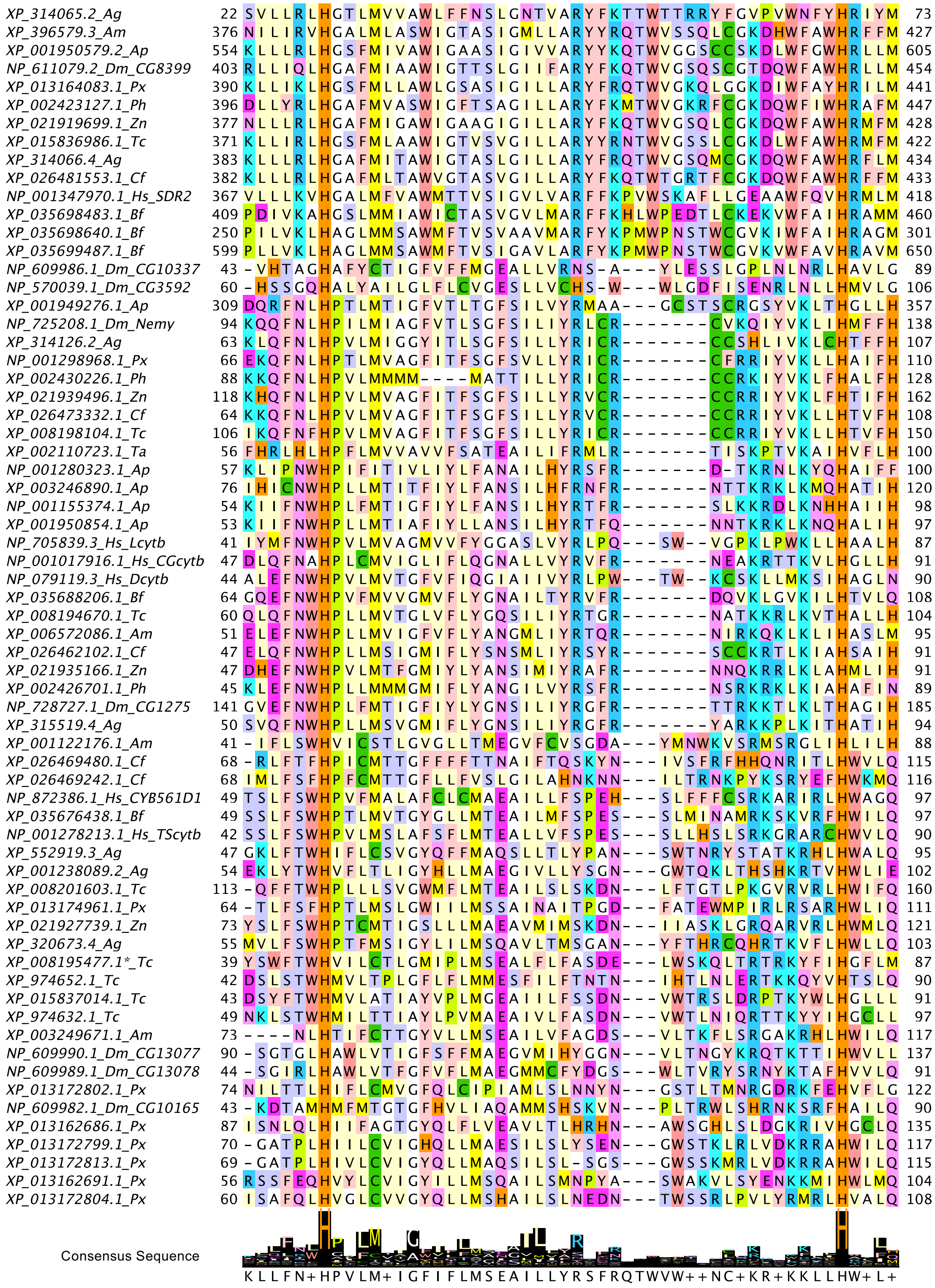
XP_314065.2_Ag 22 SVLLRLHGTLMVVAWLFFNSLGNTVARYFKTTWTTRRYFGVPVWNFYHRIYM 73
XP_396579.3_Am 376 NILIRVHGALMLASWIGTASIGMLLARYYRQTWVSSQLCGKDHWFAWHRFFM 427
XP_001950579.2_Ap 554 KLLLRLHGSFMIVAWIGAASIGIVVARYYKQTWVGGSCCSKDLWFGWHRLLM 605
NP_611079.2_Dm_CG8399 403 RLLIQLHGAFMIAAWIGTTSLGIIFARYFKQTWVGSQSCGTDQWFAWHRLLM 454
XP_013164083.1_Px 390 KLLIKLHGSFMLLAWLGSASIGILLARYFRQTWVGKQLGGKDIWFAYHRILM 441
XP_002423127.1_Ph 396 DLLYRLHGAFMVASWIGFTSAGILLARYFKMTWVGKRFCGKDQWFIWHRAFM 447
XP_021919699.1_Zn 377 NLLLRLHGAFMIGAWIGAAGIGILLARYFKQTWVGSQLCGKDQWFAWHRMFM 428
XP_015836986.1_Tc 371 KLLIRLHGSFMLAAWIGTVSVGILLARYYRNTWVGSSLCGKDLWFAWHRMFM 422
XP_314066.4_Ag 383 KLLLRLHGAFMITAWIGTASLGILLARYFRQTWVGSQMCGKDQWFAWHRFLM 434
XP_026481553.1_Cf 382 KLLLRLHGAFMLTAWVGTASVGILLARYFKQTWTGRTFCGKDQWFAWHRFFM 433
NP_001347970.1_Hs_SDR2 367 VLLLKVHGALMFVAWMTTVSIGVLVARFFKPVWSKAFLLGEAAWFQVHRMLM 418
XP_035698483.1_Bf 409 PDIVKAHGSLMMIAWICTASVGVLMARFFKHLWPEDTLCKEKVWFAIHRAMM 460
XP_035698640.1_Bf 250 PILVKLHAGLMMSAWMFTVSVAAVMARFYKPMWPNSTWCGVKIWFAIHRAGM 301
XP_035699487.1_Bf 599 PLLVKLHAGLMMSAWMFTVSIGAVLARFYKPMWPNSTWCGVKVWFAVHRAVM 650
NP_609986.1_Dm_CG10337 43 -VHTAGHAFYCTIGFVFFMGEALLVRNS-A---YLESSLGPLNLNRLHAVLG 89
NP_570039.1_Dm_CG3592 60 -HSSGQHALYAILGLFLCVGESLLVCHS-W---WLGDFISENRLNLLHMVLG 106
XP_001949276.1_Ap 309 DQRFNLHPTLMTIGFVTLTGFSILVYRMAA---GCSTSCRGSYVKLTHGLLH 357
NP_725208.1_Dm_Nemy 94 KQQFNLHPILMIAGFVTLSGFSILIYRLCR-------CVKQIYVKLIHMFFH 138
XP_314126.2_Ag 63 KLQFNLHPVLMIGGYITLSGFSILLYRICR-------CCSHLIVKLCHTFFH 107
NP_001298968.1_Px 66 EKQFNLHPTLMVAGFITFSGFSVLLYRICR-------CFRRIYVKLLHAIFH 110
XP_002430226.1_Ph 88 KKQFNLHPVLMMMM----MATTILLYRICR-------CCRKIYVKLFHALFH 128
XP_021939496.1_Zn 118 KHQFNLHPVLMVAGFITFSGFSILLYRVCR-------CCRRIYVKLFHTIFH 162
XP_026473332.1_Cf 64 KKQFNLHPVLMVAGFITFSGFSILLYRVCR-------CCRRIYVKLLHTVFH 108
XP_008198104.1_Tc 106 IKQFNFHPVLMVAGFITFSGFSILLYRICR-------CCRRIYVKLLHTVFH 150
XP_002110723.1_Ta 56 FHRLHLHPFLMVVAVVFSATEAILIFRMLR-------TISKPTVKAIHVFLH 100
NP_001280323.1_Ap 57 KLIPNWHPIFITIVLIYLFANAILHYRSFR-------D-TKRNLKYQHAIFF 100
XP_003246890.1_Ap 76 IHICNWHPLLMTITFIYLFANSILHFRNFR-------NTTKRKLKMQHATIH 120
NP_001155374.1_Ap 54 KIIFNWHPLFMTIGFIYLFANSILHYRTFR-------SLKKRDLKNHHAIIH 98
XP_001950854.1_Ap 53 KIIFNWHPLLMTIAFIYLLANSILHYRTFQ-------NNTKRKLKNQHALIH 97
NP_705839.3_Hs_Lcytb 41 IYMFNWHPVLMVAGMVVFYGGASLVYRLPQ---SW--VGPKLPWKLLHAALH 87
NP_001017916.1_Hs_CGcytb 47 DLQFNAHPLCMVIGLIFLQGNALLVYRVFR-------NEAKRTTKVLHGLLH 91
NP_079119.3_Hs_Dcytb 44 ALEFNWHPVLMVTGFVFIQGIAIIVYRLPW---TW--KCSKLLMKSIHAGLN 90
XP_035688206.1_Bf 64 GQEFNWHPVFMVVGMVFLYGNAILTYRVFR-------DQVKLGVKILHTVLQ 108
XP_008194670.1_Tc 60 QLQFNWHPLLMVTGLVFLYGQSILIYRTGR-------NATKKRLKVTHALLH 104
XP_006572086.1_Am 51 ELEFNWHPLLMVIGFVFLYANGMLIYRTQR-------NIRKQKLKLIHASLM 95
XP_026462102.1_Cf 47 ELQFNWHPLLMSIGMIFLYSNSMLIYRSYR-------SCCKRTLKIAHSAIH 91
XP_021935166.1_Zn 47 DHEFNWHPVLMTFGMIYLYANSIMIYRAFR-------NNQKRRLKLAHMLIH 91
XP_002426701.1_Ph 45 KLEFNWHPLLMMMGMIFLYANGILVYRSFR-------NSRKRKLKIAHAFIN 89
NP_728727.1_Dm_CG1275 141 GVEFNWHPLFMTIGFIYLYGNSILIYRGFR-------TTRKKTLKLTHAGIH 185
XP_315519.4_Ag 50 SVQFNWHPLLMSVGMIFLYGNSILIYRGFR-------YARKKPLKITHATIH 94
XP_001122176.1_Am 41 -IFLSWHVICSTLGVGLLTMEGVFCVSGDA---YMNWKVSRMSRGLIHLILH 88
XP_026469480.1_Cf 68 -RLFTFHPICMTTGFFFFTTNAIFTQSKYN---IVSFRFHHQNRITLHWVLQ 115
XP_026469242.1_Cf 68 IMLFSFHPFCMTTGFLLFVSLGILAHNKNN---ILTRNKPYKSRYEFHWKMQ 116
NP_872386.1_Hs_CYB561D1 49 TSLFSWHPVFMALAFCLCMAEAILLFSPEH---SLFFFCSRKARIRLHWAGQ 97
XP_035676438.1_Bf 49 SSLFSWHPTLMVTGYGLLMTEAILMFSPES---SLMINAMRKSKVRFHWILQ 97
NP_001278213.1_Hs_TScytb 42 SSLFSWHPVLMSLAFSFLMTEALLVFSPES---SLLHSLSRKGRARCHWVLQ 90
XP_552919.3_Ag 47 GKLFTWHIFLCSVGYQFFMAQSLLTLYPAN---SWTNRYSTATKRHLHWALQ 95
XP_001238089.2_Ag 54 EKLYTWHVFLTLIGYHLLMAEGIVLLYSGN---GWTQKLTHSHKRTVHWLIE 102
XP_008201603.1_Tc 113 -QFFTWHPLLLSVGWMFLMTEAILSLSKDN---LFTGTLPKGVRVRLHWIFQ 160
XP_013174961.1_Px 64 -TLFSFHPTLMSLGWIILMSSAINAITPGD---FATEWMPIRLRSARHWLIQ 111
XP_021927739.1_Zn 73 YSLFSWHPTCMTIGSLLLMAEAVMIMSKDN---IIASKLGRQARVRLHWMLQ 121
XP_320673.4_Ag 55 MVLFSWHPTFMSIGYLILMSQAVLTMSGAN---YFTHRCQHRTKVFLHWLLQ 103
XP_008195477.1*_Tc 39 YSWFTWHVILCTLGMIPLMSEALFLFASDE---LWSKQLTRTRKYFIHGFLM 87
XP_974652.1_Tc 42 DSLSTWHMVLTPLGFLFLMMESFILFTNTN---IHTLNLERTKKQYVHTSLQ 90
XP_015837014.1_Tc 43 DSYFTWHMVLATIAYVPLMGEAIILFSSDN---VWTRSLDRPTKYWLHGLLL 91
XP_974632.1_Tc 49 NKLSTWHMILTTIAYLPVMAEAIVLFASDN---VWTLNIQRTTKYYIHGCLL 97
XP_003249671.1_Am 73 ----NLHTIFCTTGYVLLMSEAILVFAGDS---VLTKFLSRGAKRHLHWILQ 117
NP_609990.1_Dm_CG13077 90 -SGTGLHAWLVTIGFSFFMAEGVMIHYGGN---VLTNGYKRQTKTTIHWVLL 137
NP_609989.1_Dm_CG13078 44 -SGIRLHAWLVTFGFVFLMAEGMMCFYDGS---WLTVRYSRNYKTAFHVVLQ 91
XP_013172802.1_Px 74 NILTTLHIFLCMVGFQLCIPIAMLSLNNYN---GSTLTMNRGDRKFEHVFLG 122
NP_609982.1_Dm_CG10165 43 -KDTAMHMFMTGTGFHVLIAQAMMSHSKVN---PLTRWLSHRNKSRFHAILQ 90
XP_013162686.1_Px 87 ISNLQLHIIFAGTGYQLFLVEAVLTLHRHN---AWSGHLSLDGKRIVHGCLQ 135
XP_013172799.1_Px 70 -GATPLHIILCVIGHQLLMAESILSLYSEN---GWSTKLRLVDKRRAHWILQ 117
XP_013172813.1_Px 69 -GATPLHIVLCVIGYQLLMAQSILSL-SGS---GWSSKMRLVDKRRAHWILQ 115
XP_013162691.1_Px 56 RSSFEQHVYLCVIGYILLMSQAILSMNPYA---SWAKVLSYENKKMIHWLMQ 104
XP_013172804.1_Px 60 ISAFQLHVGLCVVGYQLLMSHAILSLNEDN---TWSSRLPVLYRMRLHVALQ 108
Consensus Sequence
KLLFN+HPVLM+IGFIFLMSEAILLYRSFRQTWVW++NC+KR+KKLLHW+L+

S1 Fig. Part 2

```
XP_314065.2_Ag               74 IASWILTCAAIV-CIFVDVRG-----------FEAHAHSIVGLATFALVFIQ 113
XP_396579.3_Am              428 ILTWSMTIAAFV-IIFVELGEWSS-----E-----TIHASLGLATTILVFIQ 468
XP_001950579.2_Ap           606 MFTWILSLSGSA-CIFVELGEWVS-----G---PSQTHALLGVVTTVLTFFQ 648
NP_611079.2_Dm_CG8399       455 VTTWSLTVAAYV-LIWVELKQ--------A---VWHAHSIIGLITVILCFIQ 494
XP_013164083.1_Px           442 VLTWLLTMLGFI-LILVEVGGWSS-----A---GGNPHAITGIITVILCFIQ 484
XP_002423127.1_Ph           448 VSTWSLTIAAFV-MIFVEIQGWYS-----E---TSNPHAILGCITTGLAFIQ 490
XP_021919699.1_Zn           429 VLTWGLTVAAFV-LIFLELKDWSA---------EDNPHAILGCATTAQAFVQ 470
XP_015836986.1_Tc           423 VLTWALTVTGFV-LIFVELRAWSA---------EKNPHAILGTVTTIICFIQ 464
XP_314066.4_Ag              435 IVTWALTVAGIV-VIFVEIGGWSQ---------VRNPHAILGIVTTVLCFLQ 476
XP_026481553.1_Cf           434 VLTWLLTLSAFV-MIFIEIGAWSS---------EQNPHAILGTVTTILCFLQ 475
NP_001347970.1_Hs_SDR2      419 FTTTVLTCIAFV-MPFIYRGGWS---------RHAGYHPYLGCIVMTLAVLQ 460
XP_035698483.1_Bf           461 ITTVVLTIIAFI-LIMTYNN-WLW-----A--FHAGAHAIVGIIVVILALIN 503
XP_035698640.1_Bf           302 IMTVLLCVLAFV-LIFFFKE-WTF-----VTGTNAEIHAIMGIVVTFLAVAQ 346
XP_035699487.1_Bf           651 ILTVLMAVAAFV-IIFVFKE-WTF-----VTGFNATIHAVMGIIVTSLAVIQ 695
NP_609986.1_Dm_CG10337       90 LLAFLVGAGGIG-IKTWQKLERKREDPNATVRHFKSNHAFYGIIGCALLLGS 140
NP_570039.1_Dm_CG3592       107 MVGLWLGLVGIF-AKSIF-------KSKIHEPHFNSKHGLCGLLGFLLIAGA 150
XP_001949276.1_Ap           358 MGTIPCVLFGGV-AAMEYHRL-------KGLPHMYSLHSWMGLLTLMLFAIQ 401
NP_725208.1_Dm_Nemy         139 AVAIPCIALGFI-SVFASHDA-------LHKVNFYSLHSWLGFVTMGMFVLQ 182
XP_314126.2_Ag              108 ACSIPCIVIGFM-AVWDSHNQ-------QQIPNFYSLHSWLGMITMGLFALQ 151
NP_001298968.1_Px           111 ALAFPCIVIGFL-AVLDYHNK-------KGINNFYSLHSWIGLVAMGLFGLQ 154
XP_002430226.1_Ph           129 ALAIPCIVVGFL-AVWDFHNLG----Q-PPIPNFYSLHSWLGLVTCGLFILQ 174
XP_021939496.1_Zn           163 ALAIPCIVVGFI-AVLDSHNLN----PAGAIPNFYSLHSWMGLVTMGLFALQ 209
XP_026473332.1_Cf           109 ALAIPCVVVGFL-AVLDSHNLA----T-PPLPNFYSLHSWLGCVTMGLFGIQ 154
XP_008198104.1_Tc           151 ALAIPCVAVGFL-TVLDSHNLA----Q-PPIPNFYSLHSWLGLVTMGLFAIQ 196
XP_002110723.1_Ta           101 TATLALVGTALA-AVIKSKNDL-------KFTHFYSAHSWFALIVLFLYASE 144
NP_001280323.1_Ap           101 GCIIIILTIAGI-ATLGSKRFRPLFTNP-AIPNLYSLHSWLGIITMAMLLLQ 150
XP_003246890.1_Ap           121 SCIVIFGVLGIW-TGLNSN----LIANP-PIPKFYSLHSWLGIITVIMFLSQ 166
NP_001155374.1_Ap            99 GFIIVLVLLAGW-ASFASH----MYSNP-PIPDLYSLHSWLGVVTISMFLSQ 144
XP_001950854.1_Ap            98 GCILILIVIAGF-AAFVSH----QYAKP-PIPHLYSLHSWLGVLTIVMFLSQ 143
NP_705839.3_Hs_Lcytb         88 LMAFVLTVVGLV-AVFTFHNHG-------RTANLYSLHSWLGITTVFLFACQ 131
NP_001017916.1_Hs_CGcytb     92 IFALVIALVGLV-AVFDYHRKK-------GYADLYSLHSWCGILVFVLYFVQ 135
NP_079119.3_Hs_Dcytb         91 AVAAILAIISVV-AVFENHNVN-------NIANMYSLHSWVGLIAVICYLLQ 134
XP_035688206.1_Bf           109 IAALIFSIVALV-ATFDFHNAN-------GIANMYSLHSWVGLIVVILFALQ 152
XP_008194670.1_Tc           105 LSAFILAVIGLK-AAFDSHNYA-----KPPKPNLYTLHSWFGLVAVIIFTGQ 150
XP_006572086.1_Am            96 LLTIALVVISLV-AVFDSHNLN-----VKPIPNMYSLHSWIGLTSIILFCCQ 141
XP_026462102.1_Cf            92 GLIFLLTIFALI-AVFDSHNLHMKDGQSDPIPNMYSLHSWIGLTAVILFACQ 142
XP_021935166.1_Zn            92 FTTFILVIIGLV-AVFDSHNLK----NP-PMPNLYTLHSWIGLSAVILFACQ 137
XP_002426701.1_Ph            90 AMAFIFTIIGLQ-AVFDSHNLA----SPSPIPNMYSLHSWLGLASVILFALQ 136
NP_728727.1_Dm_CG1275       186 MGAFILTVIALK-TVFDSHNLA----NP-PIPNMYSLHSWLGLSAVIVFSLQ 231
XP_315519.4_Ag               95 GAAFIFTVVALV-AVFDSHNLA----KPNPIPNMYTLHSWVGLSAVILFSLQ 141
XP_001122176.1_Am            89 LIGLLLVLIGMI-MIIIGKIEY-------KKSHFVTVHGIISLISIIFAVLI 132
XP_026469480.1_Cf           116 GASVILMTIGFI-LVILTVNQQ-------GAPHFFTNHSIVGLTAYILMCVS 159
XP_026469242.1_Cf           117 LGAIIFLTIGFT-IILVNKNLY-------GKPHFTSYHGIFGLITYILLVLT 160
NP_872386.1_Hs_CYB561D1      98 TLAILCAALGLG-FIISSRTRS-------ELPHLVSWHSWVGALTLLATAVQ 141
XP_035676438.1_Bf            98 AGAVVCAVGGLM-AIYLNKERM-------GKDHFVSWHGLIGVTTVFYSVVQ 141
NP_001278213.1_Hs_TScytb     91 LLALLCALLGLG-LVILHKEQL-------GKAHLVTRHGQAGLLAVLWAGLQ 134
XP_552919.3_Ag               96 AIGCVAILVGIV-IEIYLKEDA-------GRSHFRSDHAITGLVSLIFIALS 139
XP_001238089.2_Ag           103 AVGCSCCVVGIA-LEIYFREST-------NRRHFSSTHSIVGLVSLAFLALT 146
XP_008201603.1_Tc           161 SIACVLITIGFV-VVYVNKDYL-------NKQHFKTWHGLLGLIGIVFCIPS 204
XP_013174961.1_Px           112 MLALIIILTGFF-VIFANKIIN-------EKSHFVTLHAKFGIASLVFMCIT 155
XP_021927739.1_Zn           122 AGAAAFIFSGFL-VIVINKNIH-------NKNHFQSWHSIIGLVFLVFVGFT 165
XP_320673.4_Ag              104 AVAGVLISIASV-CIFLNKVRL-------GKPHFQTLHGIFGLVTVCLTLVS 147
XP_008195477.1*_Tc           88 TLGTVLVTAGCIDTFCFIT----------EGYHLYTAHGITGLVAMILMIVS 129
XP_974652.1_Tc               91 FLSTVCITVGIS-FKISSK----------NGDHFTSTHAILGLVAWILVWCS 131
XP_015837014.1_Tc            92 FISAVLVTAGIS-LMVDHN----------HGKHFHSTHGWTGLVSWIFVLLS 132
XP_974632.1_Tc               98 FCSAVLATVGIA-FMINVH----------SNPHFHSTHAWTGLVSWILVLLS 138
XP_003249671.1_Am           118 ILGLILIVSGVV-LMCRVK----------P-VHFKSVHGILGITSTVLAVFL 157
NP_609990.1_Dm_CG13077      138 TLGGGCGAAGAL-IKMIQK----------GFLLQ-STHGRLGMTAFVLCILA 177
NP_609989.1_Dm_CG13078       92 ILGGGMGVAGCL-IQLIRD----------DWSISVTLHARLGFAAFVLCLIS 132
XP_013172802.1_Px           123 AFGIFCIIFGTI-TVLADR----------G-AE-TTTHATFGHAAAFLILLS 161
NP_609982.1_Dm_CG10165       91 IVGGSMVLLGSL-GKFSSK----------E-VHFNTWHGRVGGAASFFCAAS 130
XP_013162686.1_Px           136 LIGAIFVIAGTF-LGLAEV----------D-MQINTPHGICGVIALAFSLIS 175
XP_013172799.1_Px           118 ILGSGIALAGNF-IKIVDK----------N-VHWNTYHGQFALVAIVFTVAS 157
XP_013172813.1_Px           116 ILGSGLALAGSF-IKILDK----------T-VHWNTYHGQFALVAMVFTVAC 155
XP_013162691.1_Px           105 ILGSVLAIAGSI-VKLVDG----------V-PNFRSAHGILGTVAMLFTIAS 144
XP_013172804.1_Px           109 ILGSVLAIAGSV-IMMMEK----------S-VNFTSVHGKLALAALIFTSVS 148
```

Consensus Sequence

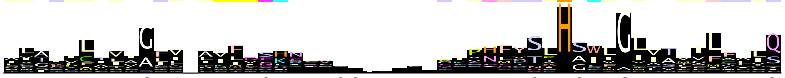

+LAIILTVIGFVDAVFDSHNLWSSYANPPPIPHFYSLHSWLGLVTVILFALQ

S1 Fig. Part 3

Sequence	Start	Alignment	End
XP_314065.2_Ag	114	PILGLMRPSQQ-P----A-----QS-AIRILHTLLGHAAYILAVTNMFLGIG	154
XP_396579.3_Am	469	PFMAAARPHPGAP----R-----RS-LFNWVHWFVGNAAHICSIIAMFFAVR	510
XP_001950579.2_Ap	649	PIFAAFRPHPDSS----K-----RP-IFNWIHWLVGNAAHIFAILTIFFAVT	690
NP_611079.2_Dm_CG8399	495	PIGALFRPGPNDK----K-----RP-YFNWGHWLGGNLAHILGIVTIFFSVK	536
XP_013164083.1_Px	485	PIGAYFRPHPGTK----K-----RP-IFNWMHWFFGNSAHILGIVTIFLAVY	526
XP_002423127.1_Ph	491	PFGAALRPSPDSP----K-----RP-IFNWLHWLVGNCAHILAIVTIFFAVK	532
XP_021919699.1_Zn	471	PFGAAFRPHPDSR----R-----RP-IFNWLHWLIGNAAHILGIVTIFFATK	512
XP_015836986.1_Tc	465	PIGAYFRPHPGTP----K-----RP-VFNWIHWLGGNVAHIIGIVTIFFAVK	506
XP_314066.4_Ag	477	PIGAFFRPHPGSS----K-----RP-IFNWLHWLGGNLAHVIAIVAIFFAVQ	518
XP_026481553.1_Cf	476	PIGAFFRPHPGTK----R-----RP-IFNWLHWLGGNSAHILAIVTIFFAVK	517
NP_001347970.1_Hs_SDR2	461	PLLAVFRPPLHDP----R-----RQ-MFNWTHWSMGTAARIIAVAAMFLGMD	502
XP_035698483.1_Bf	504	PLMALARPHPDQP----K-----RF-IFNWAHWGVGTVARILGVVAIFLGMD	545
XP_035698640.1_Bf	347	PFMSLVRGGPNEP----K-----RR-VFNWFHWAFGTGARVGAIIVMFLGLD	388
XP_035699487.1_Bf	696	PFMSLLRPGPDEP----N-----RV-VFNWFHWGFGTAARIMAIIVMFL---	734
NP_609986.1_Dm_CG10337	141	VLSGLPLYFINS------------GFALKMLHRFFGLSGFLVLMVSQMFGYN	180
NP_570039.1_Dm_CG3592	151	VASGFALVCF-T------------HLALHVIHRLMGLCGFVLLSCSQWFALN	189
XP_001949276.1_Ap	402	LILGLFTFVVLLCCRG-ATAVC-RLRCFTPIHATLGLCTFTLAIATCLTGLQ	451
NP_725208.1_Dm_Nemy	183	FVIGFFSFLVMLCCEN-KTYSC-RS-AMVPIHASLGLANFWLAIATSVTGLI	231
XP_314126.2_Ag	152	FVLGFFSFLILLCCEN-ATYKF-RS-TMVPIHASFGVATFMLAIATAVTGLT	200
NP_001298968.1_Px	155	YAVGFFSFLLLLICNK-GTAGF-RA-SLVPVHAAFGVLTFVLGVAACLTGLT	203
XP_002430226.1_Ph	175	FVVGTFSFLILLCCEG-ATAAF-RA-SLTPVHSSFGITTFMLAVATCLTGFT	223
XP_021939496.1_Zn	210	FVVGFFSFLILLCCEG-ATAAF-RA-SLVPIHATFGITTFMLAVATCLTGLT	258
XP_026473332.1_Cf	155	FLVGFFSFLLLLCCDA-ATQSF-RE-AMVPIHASFGLVTFVLAVATCLTGLT	203
XP_008198104.1_Tc	197	FVVGFFSFLILLCCEG-ATAGF-RA-SLVPIHASFGLTTFMLAIAACLTGLT	245
XP_002110723.1_Ta	145	YVAGIIVFVLPTRCIS-D--TM-RQ-NILPFHKFFGLCTYVFALITIGMGVF	191
NP_001280323.1_Ap	151	FASGFWIYLYPTV--A-A--QY-RE-IMMPYHVFFGVSSFVLAIASGGLGFC	195
XP_003246890.1_Ap	167	FTSGFISFLYPEI--G-M--RY-KK-VIMPYHIIFGILTFVLSIITSVLGFS	211
NP_001155374.1_Ap	145	FVSGFVSFLFPGI--A-A--QY-KE-AVMPYHIFFGIFNFVLAIATSVLGFS	189
XP_001950854.1_Ap	144	LFSGLWCFLYPGA--A-K--QH-RE-TLAPYHVFFGISIFILAVATAVLGFC	188
NP_705839.3_Hs_Lcytb	132	WFLGFAVFLLPWA--S-M--WL-RS-LLKPIHVFFGAAILSLSIASVISGIN	176
NP_001017916.1_Hs_CGcytb	136	WLVGFSFFLFPGA--S-F--SL-RS-RYRPQHIFFGATIFLLSVGTALLGLK	180
NP_079119.3_Hs_Dcytb	135	LLSGFSVFLLPWA--P-L--SL-RA-FLMPIHVYSGIVIFGTVIATALMGLT	179
XP_035688206.1_Bf	153	WVLGMSTYLLPFA--S-R--WI-RT-LFMPYHRFFGLVIFAMSIATCLMGLT	197
XP_008194670.1_Tc	151	FVFGFVSFLFPGL--S-R--TL-RA-AFLPVHVATGIATFTLCVVTALVGIT	195
XP_006572086.1_Am	142	WLAGFLSFLYPKL--Q-I--SL-RT-AYMPIHVYFGIAGFIGVITSCLLGLN	186
XP_026462102.1_Cf	143	LLSGFVTYLYPGL--T-S--NI-KT-TYMPVHTFFGIAIFICVVVAALLGLS	187
XP_021935166.1_Zn	138	WVSGFITFFFPGL--Q-S--PL-RA-AYMPVHVFFGLAGFIAAVIAALLGLT	182
XP_002426701.1_Ph	137	WVFGLLSFLYPTL--S-A--PL-RA-TYYPIHVFFGINAFVCASASALLGLT	181
NP_728727.1_Dm_CG1275	232	YVAGFVAFLAPGL--R-E--NY-RI-AMMPLHIYFGLFGFVLAIASALMGIT	276
XP_315519.4_Ag	142	YVFGFAAYLFPGV--R-E--QL-RA-SYMPVHVFFGVAGFVMAIAAALLGLL	186
XP_001122176.1_Am	133	AASGFVVIFDKCL--YHG--CI-RPIILKIMHSFGGIFATILLLAGLITGIY	179
XP_026469480.1_Cf	160	SLGGVATLYKHRF--R-E--WF-TARAFKIAHSSFGIITYTMGTAALCLALF	205
XP_026469242.1_Cf	161	FLLSYFTLKVKFL--F-K--SL-PKNYIMLAHVTLGIVAYAQGFADFLLGIF	206
NP_872386.1_Hs_CYB561D1	142	ALCGLCLLCPRAA--R----VS-RVARLKLYHLTCGLVVYLMATVTVLLGMY	186
XP_035676438.1_Bf	142	SMGGINLLYPKLL--K-S--WM-TLAQLKMYHATFGLLGYALACSSLALGIN	187
NP_001278213.1_Hs_TScytb	135	CSGGVGLLYPKLL--P-R--WP--LAKLKLYHATSGLVGYLLGSASLLLGMC	179
XP_552919.3_Ag	140	ILNGIAAMYTVKI--K-H--LI-KPVYVKMCHYLTGIVAFVIGMTSLALEYS	185
XP_001238089.2_Ag	147	LVNGLMALFAPEL--R-R--RI-RPIYSKLGHYLTGTVCYVLGMVAIVLAYE	192
XP_008201603.1_Tc	205	CLNGVAALYHGSL--R-Q--LV-RPKIVKFVHILTGIVSFGFGGAALILSVY	250
XP_013174961.1_Px	156	SLGGVTALYSLKL--K-D--YI-APIYTKLLHASAGLLTFTLGIITILLGTL	201
XP_021927739.1_Zn	166	SSGGVITLYSFRL--K-Q--YL-KPAKMKLIHNLIGVLTFVIGVVTEIFGIY	211
XP_320673.4_Ag	148	IAGGVTTKYAFQL--R-H--YM-RPIYSKLGHGIVGTVAYLLATVTISLGVY	193
XP_008195477.1*_Tc	130	LVLGAMANYSNDL--R-K--YA-RPVTFKFNHNFVGLVGYIIGIVSLCFSFY	175
XP_974652.1_Tc	132	VLLGLASSQSQLL--K-K--IA-KPVVLKFLHNLIGICAFTFGIITLCYGL-	176
XP_015837014.1_Tc	133	QILGLAAAKSTIF--A-K--IV-PPVWIKFLHNFLGILGYVFGIVSLAYGLE	178
XP_974632.1_Tc	139	QFLGIASAKSQDL--K-N--IL-RPVWFKFLHNLFGMLGYIFGLVSLCYGLD	184
XP_003249671.1_Am	158	SVAGYPVFIAAKL--R-S--AI-KPVAIKFAHNFLGISCFAIGMAAQCYGYT	203
NP_609990.1_Dm_CG13077	178	MSSGLAALFSSRI--K-K--LI-TPLLNKTFHNFLGFACFVIALVTQYYGYQ	223
NP_609989.1_Dm_CG13078	133	LLSGLVAFLARCL--S-R--TI-SPLVNKTFHVVLSFTAFVIAMMAQFYGYT	178
XP_013172802.1_Px	162	CMTGLCSNGQGLF-----------VIVIKYVHILTGIFGFIASSLCFTSGLY	202
NP_609982.1_Dm_CG10165	131	IVGGFVNYFQPKF--A-L--KVMPPSELRFRHNLFGLVTFSLGMGAIYLGYY	177
XP_013162686.1_Px	176	FITGIIALFSSKV--R-L--MI-KTGPVKILHIAVGLFAISMGLITMAIGFN	221
XP_013172799.1_Px	158	LINGLTSLYAYEW--R-R--LF-NGTISKLTHICFGIVTFAASSISLCYGFD	203
XP_013172813.1_Px	156	LLNGLTSLYAYEW--R-R--IF-NGSISKLTHICFGIVAFGAACISLCYGFD	201
XP_013162691.1_Px	145	LISGIVNLFSMNF--S-N--N---INIIKIVHSVLGALALSVAYLSLCFAFH	188
XP_013172804.1_Px	149	LINGTISFFSNGR--R-N--T---IKIAKMNHVLCGTAAFVISSVCLCFGYY	192

Consensus Sequence

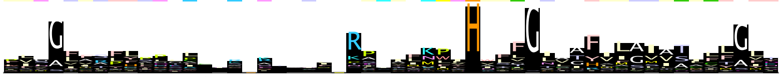

P+GGFFSFLPPLLCERHKTASLMRPVIFKPIHWFFGI+AFILAIAT+LLGLT
