# Supplementary material for: Comparison of insect and human cytochrome b561 proteins: Insights into candidate ferric reductases in insects
Source: PLoS One. 2023 Dec 1;18(12):e0291564. doi: 10.1371/journal.pone.0291564 (PMC10691727; doi:10.1371/journal.pone.0291564)

S2 Fig. Alignment of the homologous cytb561 core domain in all analyzed insect sequences.

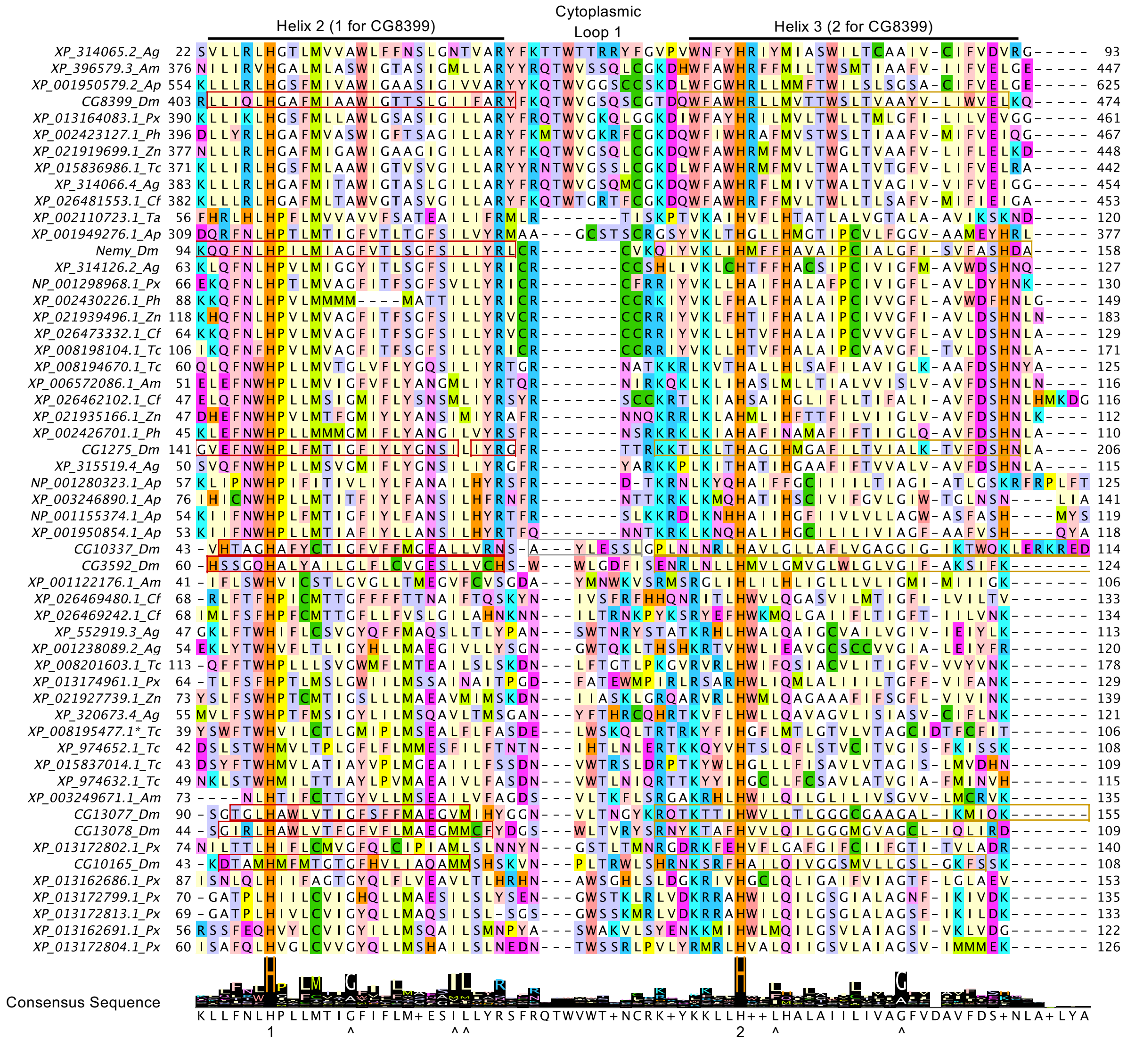

S2 Fig. continued

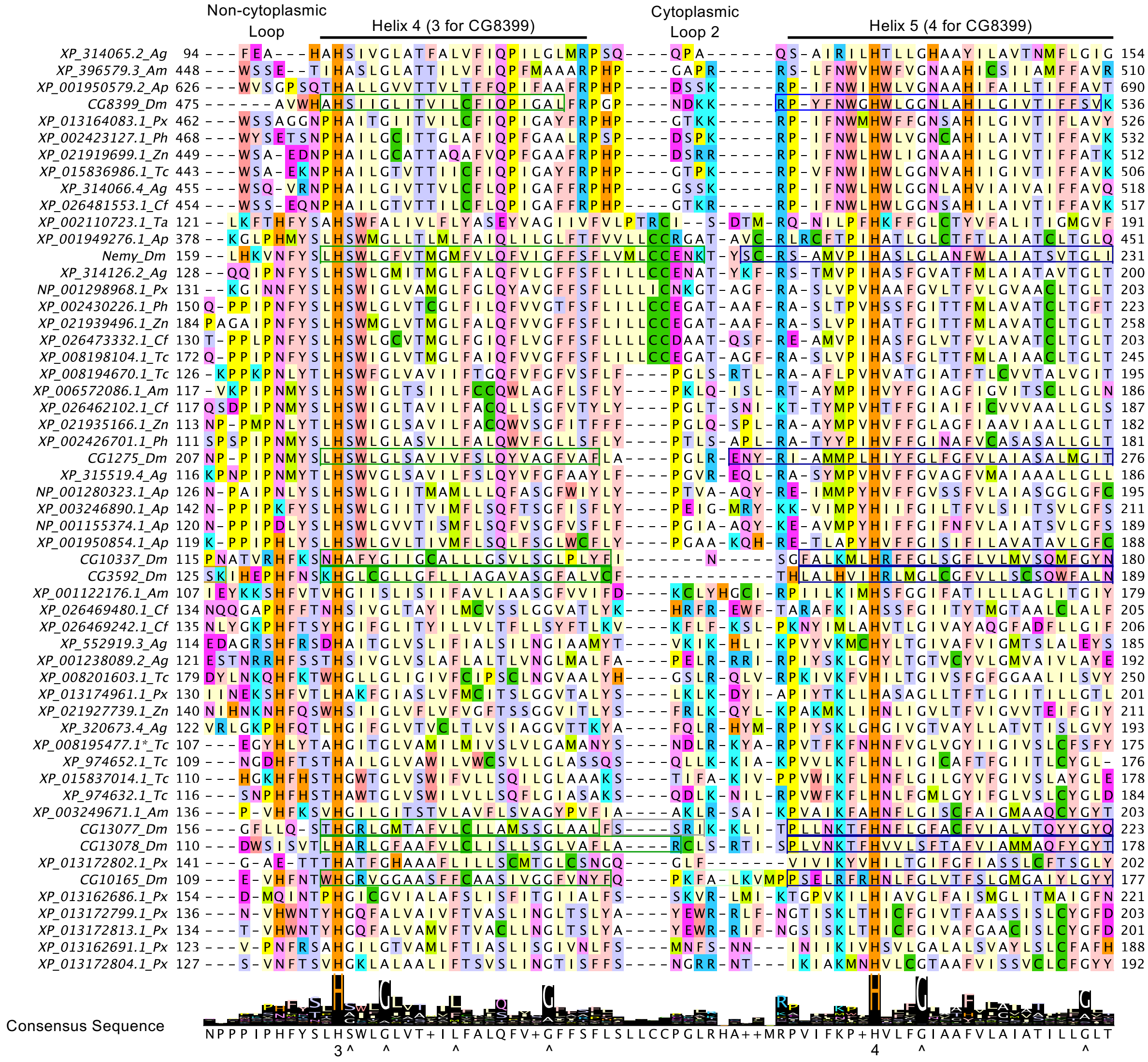

Supplement: S2 Fig — Cytb561 protein sequences from A. gambiae (Ag), A. mellifera (Am), A. pisum (Ap), C. felis (Cf), D. melanogaster (Dm), T. castaneum (Tc), P. humanus corporis (Ph), P. xuthus (Px), and Z. nevadensis (Zn) were aligned with an outgroup sequence from T. adhaerens (Ta). AlphaFold models of the D. melanogaster cytb561s were used to predict transmembrane helices and loop regions: helix 2 is boxed in red, helix 3 in yellow, helix 4 in green, and helix 5 in blue (helices 1–4 for CG8399); short helices predicted between the transmembrane helices are shown in gray. The approximate location of transmembrane helices and loops are labeled at the top of the alignment. The four strictly-conserved histidines are numbered in order of occurrence; 11 highly-conserved residues (Jalview conservation score of 8 or 9) are indicated with a caret (^). The Jalview consensus row shows the consensus logos with the most common residue at that position listed below the logo; a + is used where equal top residues were calculated by Jalview. Coloring by amino acid residue: basic residues in blue (Arg) or cyan (Lys); acidic residues (Glu and Asp) in dark pink; amide residues (Asn and Gln) in light pink; hydroxylic residues (Ser and Thr) in pale purple; aromatic residues in light peach (Phe and Tyr) or dark peach (Trp); sulfur-containing residues in dark green (Cys) or light green (Met); His in orange; Pro in yellow; Ile, Leu, and Val in pale yellow; and Ala and Gly in white. (PDF) [file pone.0291564.s002.pdf]
