# Supplementary material for: Comparison of insect and human cytochrome b561 proteins: Insights into candidate ferric reductases in insects
Source: PLoS One. 2023 Dec 1;18(12):e0291564. doi: 10.1371/journal.pone.0291564 (PMC10691727; doi:10.1371/journal.pone.0291564)
Supplement: S3 Fig — An HHPred search using the sequence of the D. melanogaster CG8399 DOMON domain matched with the P. chrysosporium cellobiose dehydrogenase (CDH) cytochrome domain (PDB 1D7B). The CDH methionine and histidine residues that bind to bind a b-type heme group in CDH are conserved in the CG8399 DOMON domain and are marked with an asterisk (*). (PDF) [file pone.0291564.s003.pdf]

S3 Fig. HHpred alignment between *D. melanogaster* CG8399 DOMON domain and *P. chrysosporium* cellobiose dehydrogenase (CDH) cytochrome domain (PDB 1D7B).

```
                                                                   *
1D7B_CDB_Pchr   24 DVTYGFVFPPLATSGAQSTEFIGEVVAPI----ASKW-IGIALG--GAMNNDLLLVAWA  75
CG8399_Dm      249 TSITVVTVRGD--------VFEFEIQSGKG--TNAA-YVAVGLSDDAKMGDDLTTECVP 296

1D7B_CDB_Pchr   76 NGN----QIVSSTRWAT-GY--V-QPTAYTGTATLTTLPETTINSTHWKWVFRCQGCTE 126
CG8399_Dm      297 ENG----RVNLYSSLTSASP-YSAVRSNVNQ-NSARLL-DASIVDGVIYCRVQRDAVTN 348

                                                        *
1D7B_CDB_Pchr  127 WNNGGGIDVTSQGVLAWAFSNVAVDDPSDPQSTFSEHTD---FGFFGIDYSTAHSAN   180
CG8399_Dm      349 VQGRTFDLRNGKYHLLVASGSSLKE------NSVGYHDIGRLPSAQPINLAEVQDLS   399
```
