# Supplementary material for: Comparison of insect and human cytochrome b561 proteins: Insights into candidate ferric reductases in insects
Source: PLoS One. 2023 Dec 1;18(12):e0291564. doi: 10.1371/journal.pone.0291564 (PMC10691727; doi:10.1371/journal.pone.0291564)

S4 Fig. Alignment of DOMON domains from insect CG8399 proteins and CYBDOMs from other diverse species.

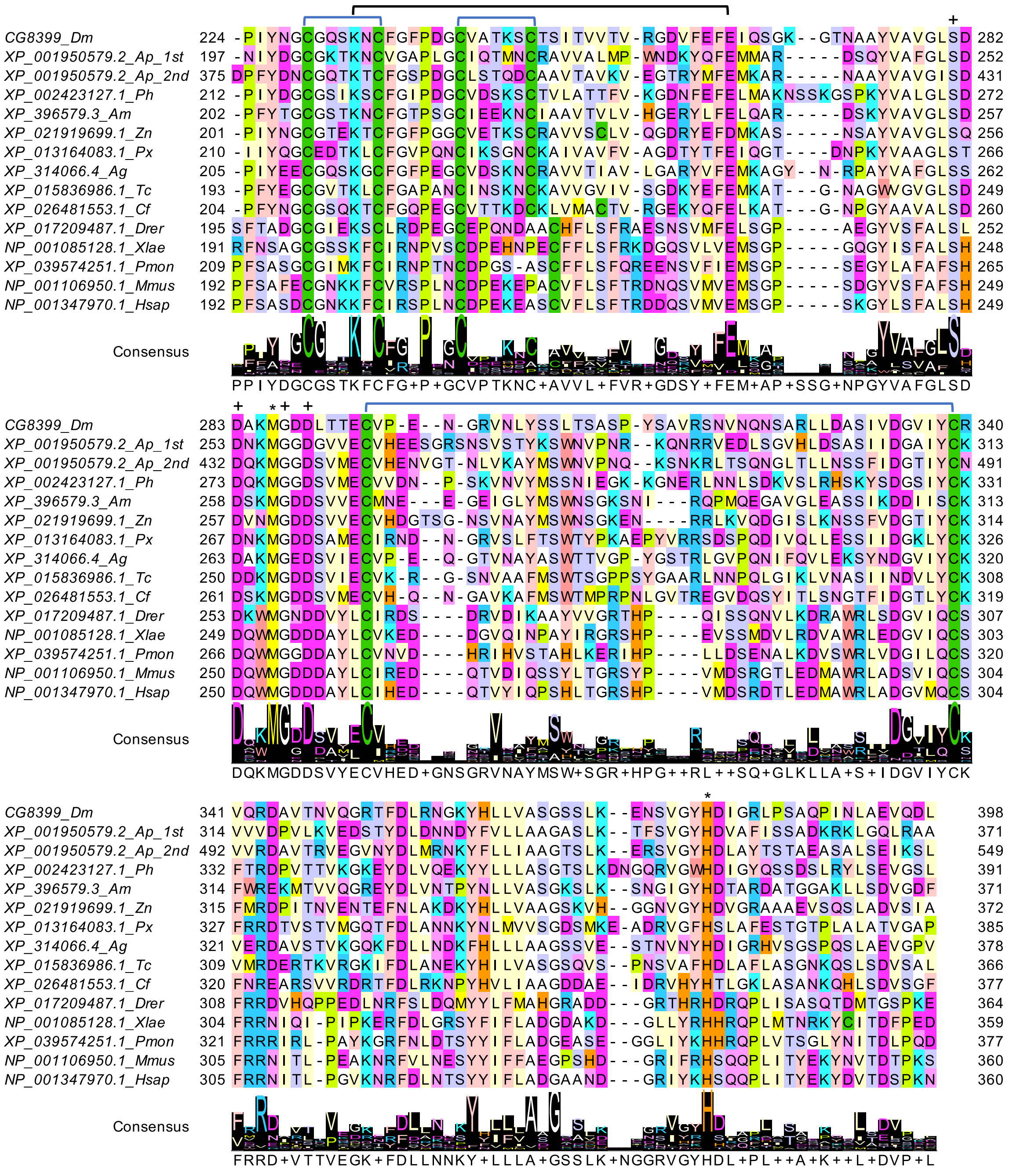

Supplement: S4 Fig — DOMON domains from insect species (as in S2 Fig, including both A. piusm DOMON domains) and non-insect animals D. rerio, (Drer, zebrafish); X. laevis (Xlae, frog); P. montanus (Pmon, sparrow); M. musculus (Mmus, mouse); H. sapiens (Hsap, human) were aligned. Conserved residues include putative heme-binding methionine and histidine residues (*), residues surrounding the conserved methionine (+), cysteines predicted to form disulfide bonds (blue brackets), and Lys233 and Glu264, which are positioned to form a salt bridge (black bracket). Alignment colored as in S2 Fig. The Jalview consensus rows show the most common residue at that position listed below the logo; a + is used where there are equal top residues. (PDF) [file pone.0291564.s004.pdf]
