# Supplementary material for: Comparison of insect and human cytochrome b561 proteins: Insights into candidate ferric reductases in insects
Source: PLoS One. 2023 Dec 1;18(12):e0291564. doi: 10.1371/journal.pone.0291564 (PMC10691727; doi:10.1371/journal.pone.0291564)
Supplement: S5 Fig — The A. pisum Nemy sequence was aligned with insect sequences exhibiting similarly extended regions in the N-terminus and the non-cytoplasmic loop connecting helix 1 and helix 2. BLAST-identified insect sequences are from: Aphis craccivora (Ac), Aphis glycines (Agl), Aphis gossypii (Ago), Cinara cedri (Cc), Diuraphis noxia (Dn), Melanaphis sacchari (Ms), Myzus persicae (Mp), Rhopalosiphum maidis (Rm), and Sipha flava (Sf). The longest D. melanogaster Nemy isoform is included for comparison. Colored as in S2 Fig, including aspartate and glutamate in magenta, glycine and alanine in white, and serine and threonine in purple. H1-H4 are indicated by asterisks. Lines above the alignment show predicted helices based on the ChimeraX structure of D. melanogaster Nemy. (PDF) [file pone.0291564.s005.pdf]

## S5 Fig. Alignment showing extended regions in the Aphid Nemy sequences

```
Nemy_A_Dm                ------------------------------------------------------------
VVC36571.1_Cc         1  ----MDSQQNTFLPPPIDGGEASAPLAQPDDDFVTANSAAGTPNTPADDAGTGSTVSPVS   56
KAE9533855.1_Agly     1  MDNQNNS-TTTFLPPPIDG-EASAPLAQPDDDFVVPLSAAGTPNTPADDGGTGSTVSPVT   58
XP_027844671.1_Ago    1  MDNQNNSTTSTFLPPPIDG-EASAPLAQPDDDFVVPLSAAGTPNTPADDGGTGSTVSPVT   59
KAF0754846.1_Ac       1  MDNQNNSNTTTFLPPPIDG-EASAPLAQPDDDFVVPLSAAGTPNTPADDGGTGSTVSPVT   59
XP_026816157.1_Rm     1  MDNQNNSTTTTFLPPPIDG-EASAPLAQPDDDFVVPLSAAGTPNTPADDGGTGSTVSPVT   59
XP_025196103.1_Ms     1  MDNQNNSTTSTFLPPPIDG-EASAPLAQPDDDFVVPLSAAGTPNTPADDGGTGSTVSPVT   59
XP_001949276.1_Ap     1  MDNQNNSTTTTFLPPPIDG-EASAPLAQPDDDFVVPLSVAGTPNTPADDGGTGSTVSPVS   59
XP_022171233.1_Mp     1  MDNQNNSNTTTFLPPPIDG-EASAPLAQPDDDFVVPLSAAGTPNTPADDGGTGSTVSPVS   59
XP_015366894.1_Dn     1  MDNQNNSTTTTFLPPPIDG-EASAPLAQPDDDFVVPLSAAGTPNTPADDGGTGSTVSPVS   59

Nemy_A_Dm                ------------------------------------------------------------
VVC36571.1_Cc        57  PVHLNRQKINAMMIDDDQVE-ADNDNGATDDSAGLLPPPDETDTDDKSTKDTSTTVTKND  115
KAE9533855.1_Agly    59  PVHLNRQPHAL-MMDDDQAANAATDSGATDDNSVLLPPPEETDSEEKPNKDGGDDGDNGG  117
XP_027844671.1_Ago   60  PVHLNRQPHAL-MMDDDQAANAATDSGATDDNSVLLPPPEETDSEEKPNKDGGDDGDNGV  118
KAF0754846.1_Ac      60  PVHLNRQPHAL-MMDDDQAANAATDSGATDDNSVLLPPPDETDGEEKPNKDGGDEEVNGG  118
XP_026816157.1_Rm    60  PVHLNRQPHGAMMMDDDQAATGAADSGATDDSSVLLPPPDETDNEEKPN-KDNADDEDNG  118
XP_025196103.1_Ms    60  PIHLGRHPHGAMMMDDDQAATAAADGGATDDSSVLLPPPDETDGEEKPNNKDNADGEDNG  119
XP_001949276.1_Ap    60  PVHLNRQQHGAMMMDDDQAANTAADSGATDDSSVLLPPPDDTENEEKPNKDNADDEDNGG  119
XP_022171233.1_Mp    60  PVHLNRQQHGAMMMDDDQAANTVAEGGATDDSSVLLPPPDETENEEKPNKDNADDEDNGG  119
XP_015366894.1_Dn    60  PVHLNRQQHGALMMDDDQAANTAADSGATDDSSVLLPPPDETDNEEKSNKDNADDEDNGG  119

Nemy_A_Dm             1  -------------------------------------------------MDTNSVSPLPP   11
VVC36571.1_Cc       116  NDAQWSEKPLGDDDDDDDQVPVLAMSGILPATATGGGGGGGGGGAGTGDSKKNVTGSNDP  175
KAE9533855.1_Agly   118  -----GDNNENRTGEDDDQVPVLAMSGILPATATGGGP--------ISDSKKNAGGPNDS  164
XP_027844671.1_Ago  119  -----EDNNENRAGEDDDQVPVLAMSGILPATATGGGP--------ISDSKKNAGGPNDS  165
KAF0754846.1_Ac     119  -----GDNNDNGAGEDDDQVPVLAMSGILPATATGGGP--------ISDSKKNAGGPNES  165
XP_026816157.1_Rm   119  -----GDNNDNRTGEDDDQVPVLAMSGILPATASGGGS--------ISDSKKNAGGPNDS  165
XP_025196103.1_Ms   120  -----GDNDDNGAAGDDDQVPVLAMSGILPATATGGGP--------ISDSKKNAGGPNDS  166
XP_001949276.1_Ap   120  -----DNN--DEAADDDDQVPVLAMSGILPATATGGGQ--------ISDSKKNAGGPNDS  164
XP_022171233.1_Mp   120  -----DNN--GEAADDDDQVPVLAMSGILPATATGGGP--------ISDSKKTAGGPNDS  164
XP_015366894.1_Dn   120  -----DNN--DEAADDDDQVPVLAMSGILPATATGGGP--------ISDSKKTSGGPNDT  164

Nemy_A_Dm            12  M---------EEAMDRVSEKSPPGTPNGIEMPPPPDEKRYEEDPDNAWNCCSWCEYLLIV   62
VVC36571.1_Cc       176  TSGGAGGTVTGNGENKSNEKSRYASGNGAGG-GTDQDDSGANNSQAPLGCGTTLEYVLLI  234
KAE9533855.1_Agly   165  LTGGAPG--TGNGENKSKEKSRYTTGSGTGA-GTDVEDSGTNNPQAALGCGTTFEYVVLV  221
XP_027844671.1_Ago  166  LTGGAPG--TGNGESKSKEKSRYTTGSGTGA-GTDVEDSGTNNPQAALGCGTTFEYVVLV  222
KAF0754846.1_Ac     166  LTGGAPG--TGNGENKSKEKSRYTAGSGTGA-GTDVEDSGTNNPQAALGCGTTFEYVVLV  222
XP_026816157.1_Rm   166  LTGGGTG--TGNGENKSKEKSRYTTGSGTGA-GTDVEDSGTNSPQAALGCGTTFEYVVLV  222
XP_025196103.1_Ms   167  LTGGGTGTGTGNGENKSKEKSRYTAGSGTGA-GTDVEDSGTSNPQAALGCGTTFEYVVLI  225
XP_001949276.1_Ap   165  A-GGGAG--TGNGENKSKEKSRYTTGSGTGA-GTDAEDSGTNNPQTALGCGTTFEYVVLV  220
XP_022171233.1_Mp   165  LTGGGTG--TGNGENKSKEKTRYTTGSGTGT-GTDAEDSGTNNPQAALGCGTTFEYVVLV  221
XP_015366894.1_Dn   165  LTGGGAG--TGNGENKSKEKTRYTAGSGTGT-GTDAEDSGTSNPQAALGCGTTFEYVVLV  221

                         Predicted Helix 1
Nemy_A_Dm            63  ILSTILLVGVLVLTLFWVMFYRDGFA-----WSE--------------------------   91
VVC36571.1_Cc       235  TTCTILLLSVTGTTIYWTMVYRGGYDCAWFPWPPQTPKQLLSVGIFTYKHHSQNSQNFSD  294
KAE9533855.1_Agly   222  TACSILLLAVTGTTVYWTMAYRGGYDPNWFPWPPQTPKQMLSVGVFTYKHP-QNFTA---  277
XP_027844671.1_Ago  223  TACSILLLAVTGTTVYWTMAYRGGYDPNWFPWPPQTPKQMLSVGVFTYKHP-QNFTA---  278
KAF0754846.1_Ac     223  TACSILLLAVTGTTVYWTMAYRGGYDPNWFPWPPQTPKQMLSVGVFTYKHP-QNFTT---  278
XP_026816157.1_Rm   223  TACSILLLAVTGTTVYWTMAYRGGYDPNWFPWPPQTPKQMLSVGVFTYKHP-QNFTA---  278
XP_025196103.1_Ms   226  TACSILLLAVTGTTVYWTMAYRGGYDPNWFPWPPQTPKQMLSVGVFTYKHP-QNFTT---  281
XP_001949276.1_Ap   221  TACSFLLVAVTGTTVYWTMAYRGGYDPNWFPWPPQTPLQMLTIGVFTYKHP-QNITD---  276
XP_022171233.1_Mp   222  TACSILLLAVTGTTVYWTMAYRGGYDTNWFPWPPLTPKQMLSGGVFTYKHPPQNFTT---  278
XP_015366894.1_Dn   222  TACSILLLAVTGTTIYWTMAYRGGYDTNWFPWPPLTPREMLSGGVFTYKHP-QNFTT---  277

                                                                 Predicted Helix 2
Nemy_A_Dm            92  ----------------------------------------------SPKQQFNLHPILMI  105
VVC36571.1_Cc       295  TNVDFGAGNSFGESFDGNYSTTSTIATSTPEQLSTQYTNKSSKIDLTDEQRFNLHPTLMT  354
KAE9533855.1_Agly   278  GGSDI--------------STTTIATTTVEQQQQSSQLNTSTSNVMIEDRRFNLHPTLMT  323
XP_027844671.1_Ago  279  GGSDI--------------STTTIATTTVEQQQQSSQLNTSTSNVLIEDRRFNLHPTLMT  324
KAF0754846.1_Ac     279  GSSDI--------------STTTIATTTVEQQQQSSQLNISTSNVLIEDRRFNLHPTLMT  324
XP_026816157.1_Rm   279  SGSDI--------------STTTIATTTTTEQQQSSQLNISSLNILIEDRRFNLHPTLMT  324
XP_025196103.1_Ms   282  SGSDI--------------STTTVTT-TTVEQQQSSQLNISSSNSLMEDRRFNLHPTLMT  326
XP_001949276.1_Ap   277  GGSDI--------------STTIS--TTTVEQQQSSLLNISSSNVSIEDQRFNLHPTLMT  320
XP_022171233.1_Mp   279  SASDI--------------STTVA--TTTVEQQQSSQLNISSSNFSIEDRRFNLHPTLMT  322
XP_015366894.1_Dn   278  GGSDI--------------STTIT--TTTVEQQQSSQLNISSSNVSIEDRRFNLHPTLMT  321
                                                                          *
```

## S5 Fig. continued

```
                          Predicted Helix 2                  Predicted Helix 3
Nemy_A_Dm            106 AGFVTLSGFSILIYRL---CR-CVKQIYVKLIHMFFHAVAIPCIALGFISVFASHDALHK 161
VVC36571.1_Cc        355 FGFITFTGFSILVYRMAAGCSTTCRGTYVKLTHSLLHLATVPCVLFGSVASMEYHRSKGI 414
KAE9533855.1_Agly    324 V--------AILVYRMAAGCSTSCRGTYVKLTHGLLHLATVPCVVLGAVAAMEYHRLKGL 375
XP_027844671.1_Ago   325 VGFVTLTGFSILVYRMAAGCSTSCRGTYVKLTHGLLHLATVPCVVLGAVAAMEYHRLKGL 384
KAF0754846.1_Ac      325 V--------AILVYRMAAGCSTSCRGTYVKLTHGLLHLATVPCVVLGAVAAMEYHRLKGL 376
XP_026816157.1_Rm    325 VGFVTLTGFSILVYRMAAGCSTSCRGTYVKLTHGLLHLATVPCVVLGAVAAMEYHRLKGL 384
XP_025196103.1_Ms    327 VGFVTLTGFSILVYRMAAGCSTSCRGTYVKLTHGLLHLATVPCVVLGAVAAMEYHRLKGL 386
XP_001949276.1_Ap    321 IGFVTLTGFSILVYRMAAGCSTSCRGSYVKLTHGLLHMGTIPCVLFGGVAAMEYHRLKGL 380
XP_022171233.1_Mp    323 IGLVTLTGFSILVYRMAAGCSTSCRGTYVKLTHILLHIATAPCILLGAVAAMEYHRLKGL 382
XP_015366894.1_Dn    322 IGLVTLTGFSILVYRMAAGCSTSCRGTYVKLTHVLLHIATAPCILLGAVAAMEYHRLKGL 381
                                                          *

                                      Predicted Helix 4                Predicted Helix 5
Nemy_A_Dm            162 VNFYSLHSWLGFVTMGMFVLQFVIGFFSFLVMLCCENKTYSCRS-AMVPIHASLGLANFW 220
VVC36571.1_Cc        415 PHLYSLHSWMGVLTVSLFIIQFTLGIFTFVVLLCCRGATAACRLRCFAPIHATLGLCTFT 474
KAE9533855.1_Agly    376 PHLYSLHSWMGLLTVSLFVIQFTLGLFTFVVLLCCRGATAACRLRCFAPIHATLGLCTFT 435
XP_027844671.1_Ago   385 PHLYSLHSWMGLLTVSLFVIQFTLGLFTFVVLLCCRGATAACRLRCFAPIHATLGLCTFT 444
KAF0754846.1_Ac      377 PHLYSLHSWMGLLTVSLFVIQFTLGLFTFVVLLCCRGATAACRLRCFAPIHATLGLCTFT 436
XP_026816157.1_Rm    385 PHLYSLHSWMGLLTVSLFVIQFTLGLFTFVVLLCCRGATAVCRLRCFAPIHATLGLCTFT 444
XP_025196103.1_Ms    387 PHLYSLHSWMGLLTVSLFVIQFTLGLFTFVVLLCCRGATAACRLRCFAPIHATLGLCTFT 446
XP_001949276.1_Ap    381 PHMYSLHSWMGLLTLMLFAIQLILGLFTFVVLLCCRGATAVCRLRCFTPIHATLGLCTFT 440
XP_022171233.1_Mp    383 PHMYSLHSWMGLLTFILFTIQFILGFFTFVLLLCCRGATAVCRLRCFAPIHATLGLCTFT 442
XP_015366894.1_Dn    382 PHMYSLHSWMGLLTFILFTIQFILGFFTFVLLLCCRGATAVCRLRCFAPIHATLGLCTFT 441
                               *                                           *

Nemy_A_Dm            221 LAIATSVTGLIEKERETVNEAGVSSENKLVEH---------------------------- 252
VVC36571.1_Cc        475 LAIATCLTGLQQRADFMIFNNNGS------------------------------------ 498
KAE9533855.1_Agly    436 LAIATCLTGLQQRADFSIFSNNNGQQKQHQQQQITLPPTASSSPSSLNSGELHGRALPQQ 495
XP_027844671.1_Ago   445 LAIATCLTGLQQRADFSIFSNNNG------------------------------------ 468
KAF0754846.1_Ac      437 LAIATCLTGLQQRADFSIFSNNNGN----------------------------------- 461
XP_026816157.1_Rm    445 LAIATCLTGLQQRADFSIFSNNNG------------------------------------ 468
XP_025196103.1_Ms    447 LAIATCLTGLQQRADFSIFSNNNG------------------------------------ 470
XP_001949276.1_Ap    441 LAIATCLTGLQQRADFSIFSNNNG------------------------------------ 464
XP_022171233.1_Mp    443 LAIATCLTGLQQRADFSIFSNNNG------------------------------------ 466
XP_015366894.1_Dn    442 LAIATCLTGLQQRADFSIFSNNNG------------------------------------ 465

Nemy_A_Dm                ------------------------------------------------------------
VVC36571.1_Cc            ------------------------------------------------------------
KAE9533855.1_Agly    496 AIINVLGVLLMMALVFVTVALLSQKKSCRRNSSSSLSPSTMRYCRNGGQSFQLVPSSPAA 555
XP_027844671.1_Ago       ------------------------------------------------------------
KAF0754846.1_Ac      462 -------------------------------SSSSLSPSTMRYCRNGGQSFQLVPSSPAA 490
XP_026816157.1_Rm        ------------------------------------------------------------
XP_025196103.1_Ms        ------------------------------------------------------------
XP_001949276.1_Ap        ------------------------------------------------------------
XP_022171233.1_Mp        ------------------------------------------------------------
XP_015366894.1_Dn        ------------------------------------------------------------

                                                            Predicted Helix 6
Nemy_A_Dm            253 ---------------------------------YITSAIGVTLIFIGIIVTFAVRRSNAP 279
VVC36571.1_Cc        499 ----------------AIESETIKQKLHVNKEPVVVNILAVLLVFVFIVVSYTVRRESIK 542
KAE9533855.1_Agly    556 ADGSDHGGRNGRM----------------------------------------------- 568
XP_027844671.1_Ago   469 -------------DGKQTISELIKQKLHLNKEPVVVNVLAVLLVSVLVIVSYTVRRESLK 515
KAF0754846.1_Ac      491 ADASDHGGRNGRIDGKQAISELIKQKLHLNKEPVVVNVLAVLLVSVLVIVSYTIRRESLK 550
XP_026816157.1_Rm    469 -------------DSKQTISELIKQKLHLNKEPVVVNVLAVLLVSVLVIVSYTIRRESLK 515
XP_025196103.1_Ms    471 -------------DGKQTISELIKQKLHLNKEPVVVNVLAVLLVSVLIIVSYTIRRESLK 517
XP_001949276.1_Ap    465 -------------GGEQTLSELIKQKLHLNKEPVVVNVLAVLLVSVLVIVSYTVRRESLK 511
XP_022171233.1_Mp    467 -------------GGEKTLSELIKQKLYLNKEPVVVNVLAVLLVSVLIIVSYMVRRESLK 513
XP_015366894.1_Dn    466 -------------DGEQTLSELIKKKLYLNKEPVVVNVLAVLLVSVLIVVSYMVRRESLK 512

Nemy_A_Dm            280 ASA---KVYVT----ERI------------------------------- 290
VVC36571.1_Cc        543 RKSNNKSPYTATMSPLRVKSS-SSTGKFGSVKQSSLPPV---------- 580
KAE9533855.1_Agly        -------------------------------------------------
XP_027844671.1_Ago   516 RRPNNKPSYTATVSPLRIKSSSSSTGKFGSGKQSSQPPV---------- 554
KAF0754846.1_Ac      551 RRPNNKPSYTATVSPLRIKSSSSSTGKFGSGKQSSQPPYTNTLTFIGPV 599
XP_026816157.1_Rm    516 RRPNNKPSYTATVSPLRIKSSSSSTGKFGSGKQSSQPPV---------- 554
XP_025196103.1_Ms    518 RRPNNKPSYTATVSPLRIKSSSSSTGKFGSGKQSSQPPV---------- 556
XP_001949276.1_Ap    512 RRPNNKPSYTATVSPLRVKSSSSSTGKFGTGKQSSQPPV---------- 550
XP_022171233.1_Mp    514 RRPNNKPSYTATVSPLRIKSSSSSTGKFGSGKQSSQPPV---------- 552
XP_015366894.1_Dn    513 RRPNNKPSYTATVSPLRIKSSSSSTGKFGSGKQSSQPPV---------- 551
```
