# Supplementary material for: Comparison of insect and human cytochrome b561 proteins: Insights into candidate ferric reductases in insects
Source: PLoS One. 2023 Dec 1;18(12):e0291564. doi: 10.1371/journal.pone.0291564 (PMC10691727; doi:10.1371/journal.pone.0291564)
Supplement: S1 Table — (DOCX) [file pone.0291564.s006.docx]

**S1 Table. Insect sequences used for this study.**

| **Order**  ***Species***  **(common name)** | **Accession**  **number** | **Source/**  **“Query”** | **Common homologous sequence**^1^ | **Group**^2^ |
| --- | --- | --- | --- | --- |
| Diptera  *Drosophila melanogaster*  (fruit fly) | NP_728727.1 (CG1275 isoform A) | FlyBase/  “Cytochrome b561” | G141-T276 | CG1275 |
|  | NP_725208.1 (Nemy isoform A) | FlyBase/  “Cytochrome b561” | K94-I231 | Nemy |
|  | NP_611079.2  (CG8399 isoform A) | FlyBase/  “Cytochrome b561” | R403-K536 | CG8399 |
|  | NP_609982.1  (CG10165) | FlyBase/  “Cytochrome b561” | K43-Y177 | Group 4B |
|  | NP_609990.1 (CG13077 isoform A) | FlyBase/  “Cytochrome b561” | S90-Q223 | Group 4B |
|  | NP_609989.1  (CG13078) | FlyBase/  “Cytochrome b561” | S44-T178 | Group 4B |
|  | NP_609986.1  (CG10337) | FlyBase/  “Cytochrome b561” | V43-N180 | Group 4B |
|  | NP_570039.1  (CG3592) | FlyBase/  “Cytochrome b561” | H60-N189 | Group 4B |
| Diptera  *Anopheles gambiae*  (mosquito) | XP_315519.4 | NCBI BLAST/ “NP_728727.1” | S50-L186 | CG1275 |
|  | XP_314126.2 | NCBI BLAST/  “NP_725208.1” | K63-T200 | Nemy |
|  | XP_314065.2 | NCBI BLAST/  “NP_611079.2” | S22-G154 | CG8399 |
|  | XP_314066.4 | NCBI BLAST/  “NP_611079.2” | K383-Q518 | CG8399 |
|  | XP_320673.4 | NCBI BLAST/  “NP_728727.1” | M55-Y193 | Group 4A |
|  | XP_001238089.2 | NCBI BLAST/  “NP_609982.1” | E54-E192 | Group 4B |
|  | XP_552919.3 | NCBI BLAST/  “NP_609990.1” | G47-S185 | Group 4B |
| Hemiptera  *Acyrthosiphon pisum*  (aphid) | NP_001155374.1 | NCBI BLAST/  “NP_728727.1” | K54-S189 | CG1275 |
|  | NP_001280323.1 | NCBI BLAST/  “NP_728727.1” | K57-C195 | CG1275 |
|  | XP_001950854.1 | NCBI BLAST/  “NP_728727.1” | K53-C188 | CG1275 |
|  | XP_003246890.1 | NCBI BLAST/  “NP_728727.1” | I76-S211 | CG1275 |
|  | XP_001949276.1 | NCBI BLAST/  “NP_725208.1” | D309-Q451 | Nemy |
|  | XP_001950579.2 | NCBI BLAST/  “NP_611079.2” | K554-T690 | CG8399 |
| Hymenoptera  *Apis mellifera*  (bee) | XP_006572086.1 | NCBI BLAST/  “NP_728727.1” | E51-N186 | CG1275 |
|  | XP_396579.3 | NCBI BLAST/  “NP_611079.2” | N376-R510 | CG8399 |
|  | XP_003249671.1 | NCBI BLAST/  “NP_609990.1” | N73-T203 | Group 4B |
|  | XP_001122176.1 | NCBI CDD/  “A. mellifera" + “cytochrome_b_N” | I41-Y179 | Group 4A |
| Siphonaptera  *Ctenocephalides felis*  (flea) | XP_026462102.1 | NCBI BLAST/  “NP_728727.1” | E47-S187 | CG1275 |
|  | XP_026473332.1 | NCBI BLAST/  “NP_725208.1” | K64-T203 | Nemy |
|  | XP_026481553.1 | NCBI BLAST/  “NP_611079.2” | K382-K517 | CG8399 |
|  | XP_026469480.1 | NCBI BLAST/  “NP_609990.1” | R68-F205 | Group 4A |
|  | XP_026469242.1 | NCBI CDD/  “C. felis” + “cyt_b561” | I68-F206 | Group 4A |
| Lepidoptera  *Papilio xuthus*  (butterfly) | NP_001298968.1 | NCBI BLAST/  “NP_725208.1” | E66-T203 | Nemy |
|  | XP_013164083.1 | NCBI BLAST/  “NP_611079.2” | K390-Y526 | CG8399 |
|  | XP_013172799.1 | NCBI BLAST/  “NP_609982.1” | G70-D203 | Group 4B |
|  | XP_013172813.1 | NCBI BLAST/  “NP_609982.1” | G69-D201 | Group 4B |
|  | XP_013162691.1 | NCBI BLAST/  “NP_609990.1” | R56-H188 | Group 4B |
|  | XP_013174961.1 | NCBI CDD/  “P. xuthus” + “cyt_b561” | T64-L201 | Group 4A |
|  | XP_013162686.1 | NCBI CDD/  “P. xuthus" + “cytochrome_b_N” | I87-N221 | Group 4B |
|  | XP_013172802.1 | NCBI CDD/  “P. xuthus" + “cytochrome_b_N” | N74-Y202 | Group 4B |
|  | XP_013172804.1 | NCBI CDD/  “P. xuthus" + “cytochrome_b_N” | I60-Y192 | Group 4B |
| Psocodea  *Pediculus humanus corporis*  (louse) | XP_002426701.1 | NCBI BLAST/  “NP_728727.1” | K45-T181 | CG1275 |
|  | XP_002430226.1 | NCBI BLAST/  “NP_725208.1” | K88-T223 | Nemy |
|  | XP_002423127.1 | NCBI BLAST/  “NP_611079.2” | D396-K532 | CG8399 |
| Coleoptera  *Tribolium castaneum*  (beetle) | XP_008194670.1 | NCBI BLAST/  “NP_728727.1” | Q60-T195 | CG1275 |
|  | XP_008198104.1 | NCBI BLAST/  “NP_725208.1” | I106-T245 | Nemy |
|  | XP_015836986.1 | NCBI BLAST/  ”NP_611079.2” | K371-K506 | CG8399 |
|  | XP_015837014.1 | NCBI BLAST/  “NP_609982.1” | D43-E178 | Group 4B |
|  | XP_008201603.1 | NCBI BLAST/  “NP_609990.1” | Q113-Y250 | Group 4A |
|  | XP_974632.1 | NCBI CDD/  “T. castaneum” + “cyt_b561” | N49-D184 | Group 4B |
|  | XP_974652.1 | NCBI CDD/  “T. castaneum" + “cytochrome_b_N” | D42-L176 | Group 4B |
|  | XP_008195477.1  (edited) | NCBI CDD/  “T. castaneum" + “cytochrome_b_N” | Y39-Y175 | Group 4B |
| Blattodea  *Zootermopsis nevadensis*  (termite) | XP_021935166.1 | NCBI BLAST/  “NP_728727.1” | D47-T182 | CG1275 |
|  | XP_021939496.1 | NCBI BLAST/  “NP_725208.1” | K118-T258 | Nemy |
|  | XP_021919699.1 | NCBI BLAST/  “NP_611079.2” | N377-K512 | CG8399 |
|  | XP_021927739.1 | NCBI CDD/  “Z. nevadensis” + “cyt_b561” | Y73-Y211 | Group 4A |

^1^The common homologous sequence is the amino acid sequence that corresponds to human Dcytb A44-T179. This region includes most of the core cytb561 domain of the protein.

^2^Sequences were assigned to a group based on phylogenetic and sequence analyses. The *D. melanogaster* protein name was used for all insect orthologs that group with CG1275, Nemy, or CG8399. Seven Group 4 sequences are more similar to human TScytb than any *D. melanogaster* cytb561s and are referred to as subgroup 4A, whereas the remaining Group 4 proteins belong to subgroup 4B. (Note that the 4A and 4B subgroups are based on sequence comparisons rather than phylogenetic relationship.)
