# Supplementary material for: Comparison of insect and human cytochrome b561 proteins: Insights into candidate ferric reductases in insects
Source: PLoS One. 2023 Dec 1;18(12):e0291564. doi: 10.1371/journal.pone.0291564 (PMC10691727; doi:10.1371/journal.pone.0291564)
Supplement: S2 Table — (DOCX) [file pone.0291564.s007.docx]

**S2 Table. Sequence identity between insect and *D. melanogaster* cytb561s.**

|  | | | ***D. melanogaster* Sequences** | | | | | | | |
| --- | --- | --- | --- | --- | --- | --- | --- | --- | --- | --- |
| **Species** | **Group** | **Accession Number** | **CG1275** | **Nemy** | **CG8399** | **CG10165** | **CG13077** | **CG13078** | **CG10337** | **CG3592** |
| *Dm* | CG1275 | NP_728727.1 | **100^1^** | 39.3 | 18.9 | 17 | 16.3 | 16.9 | 20.3 | 19.6 |
| *Ag* | CG1275 | XP_315519.4 | **68.6** | 35.5 | 17.4 | 16.9 | 16.2 | 16.1 | 16 | 17.9 |
| *Ap* | CG1275 | NP_001155374.1 | **54.4** | 42.1 | 14.7 | 21.3 | 17 | 15.5 | 16.8 | 18 |
| *Ap* | CG1275 | NP_001280323.1 | **43.6** | 31.9 | 15 | 16.6 | 14.5 | 13.7 | 21.7 | 15.5 |
| *Ap* | CG1275 | XP_001950854.1 | **48.5** | 33.6 | 19.6 | 15.6 | 16.3 | 17.6 | 16.8 | 17.4 |
| *Ap* | CG1275 | XP_003246890.1 | **48.5** | 33.6 | 16 | 17 | 15.6 | 18.3 | 18.1 | 16.5 |
| *Am* | CG1275 | XP_006572086.1 | **54.4** | 36.4 | 18.2 | 17 | 19.1 | 19.7 | 19.4 | 20.1 |
| *Cf* | CG1275 | XP_026462102.1 | **51.8** | 30.3 | 17.6 | 17.8 | 16.4 | 16.3 | 18.2 | 16.8 |
| *Ph* | CG1275 | XP_002426701.1 | **58.4** | 36.2 | 16.4 | 16 | 16 | 18.6 | 17.5 | 19.9 |
| *Tc* | CG1275 | XP_008194670.1 | **56.6** | 38.6 | 18.2 | 19.1 | 14.9 | 17.7 | 16.8 | 20.3 |
| *Zn* | CG1275 | XP_021935166.1 | **58.8** | 34.3 | 18.9 | 21.3 | 17 | 18.3 | 16.1 | 21 |
| *Dm* | Nemy | NP_725208.1 | 39.3 | **100** | 13.1 | 17 | 19.1 | 18.3 | 14.3 | 17.6 |
| *Ag* | Nemy | XP_314126.2 | 42.1 | **67.4** | 19.3 | 16.3 | 15.6 | 16.9 | 13.6 | 18.3 |
| *Ap* | Nemy | XP_001949276.1 | 36.6 | **46.9** | 22.6 | 14.8 | 19 | 20.3 | 17.4 | 22.2 |
| *Cf* | Nemy | XP_026473332.1 | 45 | **63.6** | 15.6 | 16.8 | 18.2 | 22.9 | 17.7 | 20.4 |
| *Px* | Nemy | NP_001298968.1 | 42.1 | **54.3** | 14.5 | 17.7 | 18.4 | 20.4 | 17 | 19 |
| *Ph* | Nemy | XP_002430226.1 | 41.4 | **52.9** | 16.3 | 14.7 | 16.8 | 19.4 | 15 | 14.8 |
| *Tc* | Nemy | XP_008198104.1 | 45.7 | **61.4** | 17 | 18.2 | 16.1 | 20.1 | 18.4 | 20.4 |
| *Zn* | Nemy | XP_021939496.1 | 42.6 | **62.4** | 16.7 | 15.1 | 15.8 | 19 | 16.3 | 19.3 |
| *Dm* | CG8399 | NP_611079.2 | 18.9 | 13.1 | **100** | 12.1 | 12.1 | 13.4 | 11.6 | 11.3 |
| *Ag* | CG8399 | XP_314065.2 | 18.1 | 13.7 | **40.3** | 14.1 | 14.1 | 16.9 | 13.5 | 12.6 |
| *Ag* | CG8399 | XP_314066.4 | 19.4 | 18.5 | **65.4** | 13.6 | 17.1 | 20.7 | 12.2 | 13.3 |
| *Ap* | CG8399 | XP_001950579.2 | 15.6 | 16.8 | **54.7** | 11.3 | 13.4 | 14.7 | 10.9 | 15.3 |
| *Am* | CG8399 | XP_396579.3 | 14.7 | 15.2 | **53.3** | 12.2 | 12.2 | 19.3 | 9.5 | 9.2 |
| *Cf* | CG8399 | XP_026481553.1 | 20.1 | 19.2 | **62.5** | 11.4 | 12.9 | 15.7 | 12.2 | 11.9 |
| *Px* | CG8399 | XP_013164083.1 | 16.8 | 15.9 | **62** | 14.9 | 12.1 | 13.4 | 15 | 9.9 |
| *Ph* | CG8399 | XP_002423127.1 | 18.9 | 16.7 | **62** | 9.7 | 11 | 13.7 | 10.9 | 10.2 |
| *Tc* | CG8399 | XP_015836986.1 | 16 | 17.1 | **65.4** | 12.1 | 12.1 | 16.4 | 10.8 | 10.5 |
| *Zn* | CG8399 | XP_021919699.1 | 19.4 | 16.4 | **64** | 10.7 | 12.1 | 15.7 | 10.8 | 9.8 |
| *Dm* | Group 4B | NP_609982.1 | 16.9 | 16.9 | 12 | **100** | 25.9 | 27.9 | 21.9 | 19.3 |
| *Dm* | Group 4B | NP_609990.1 | 16.2 | 19 | 12 | 25.9 | **100** | 51.1 | 26 | 20.7 |
| *Dm* | Group 4B | NP_609989.1 | 16.8 | 18.2 | 13.3 | 27.9 | 51.1 | **100** | 22.4 | 17 |
| *Dm* | Group 4B | NP_609986.1 | 20.1 | 14.2 | 11.5 | 21.9 | 26 | 22.4 | **100** | 34.1 |
| *Dm* | Group 4B | NP_570039.1 | 19.4 | 17.5 | 11.2 | 19.3 | 20.7 | 17 | 34.1 | **100** |
| *Ag* | Group 4A | XP_320673.4^1^ | 22.9 | 19.4 | 21.5 | **27.3** | 20.3 | 20.3 | 17.8 | 15.8 |
| *Ag* | Group 4B | XP_001238089.2 | 19 | 16.1 | 18.9 | **30.2** | 27.5 | 23.9 | 18.5 | 18 |
| *Ag* | Group 4B | XP_552919.3 | 16.2 | 14.1 | 13.3 | 27.3 | **29** | 26.1 | 22.8 | 22.5 |
| *Am* | Group 4A | XP_001122176.1^2^ | 19 | 16.7 | 14 | 20.1 | 19.4 | 19.4 | 17.1 | **21.6** |
| *Am* | Group 4B | XP_003249671.1 | 21.8 | 20.1 | 14 | 28.1 | 31.9 | **34.1** | 26.2 | 21 |
| *Cf* | Group 4A | XP_026469480.1^2^ | 21.3 | 16.8 | 16.2 | **21.6** | 21 | 15.9 | 18.5 | 16.5 |
| *Cf* | Group 4A | XP_026469242.1^2^ | **22** | 20.4 | 17.6 | 21.6 | 19.4 | 16.4 | 17.9 | 14.5 |
| *Px* | Group 4A | XP_013174961.1^2^ | 21.8 | 21.5 | 17.5 | **24.5** | 19.6 | 23.2 | 15.8 | 14.4 |
| *Px* | Group 4B | XP_013172799.1 | 15.6 | 15.5 | 10.6 | **28.1** | 27.6 | 25.9 | 20.7 | 18 |
| *Px* | Group 4B | XP_013172813.1 | 17.9 | 17 | 10.6 | **28.1** | 26.1 | 26.7 | 18.6 | 16.5 |
| *Px* | Group 4B | XP_013162691.1 | 17.6 | 18.3 | 16.8 | 24.4 | **24.6** | 24.4 | 20.5 | 18.7 |
| *Px* | Group 4B | XP_013162686.1 | 13.4 | 12 | 17.6 | **24.4** | 23.1 | 22.2 | 17.8 | 17.1 |
| *Px* | Group 4B | XP_013172802.1 | 15 | 19.7 | 9.4 | 20.9 | 19.4 | **21.4** | 19.7 | 16.1 |
| *Px* | Group 4B | XP_013172804.1 | 16.9 | 18.3 | 16.9 | 24.4 | 25.4 | **27.4** | 21.2 | 16.4 |
| *Tc* | Group 4A | XP_008201603.1^2^ | 21.3 | 19.6 | 12.7 | **25.9** | 20.9 | 19.4 | 19.3 | 17.4 |
| *Tc* | Group 4B | XP_008195477.1^3^ | 17.5 | 16.9 | 18.1 | 22.6 | **27.9** | 24.1 | 20 | 17.9 |
| *Tc* | Group 4B | XP_015837014.1 | 20.4 | 16.2 | 16.8 | 24.3 | **30.4** | 22.2 | 22.8 | 17.9 |
| *Tc* | Group 4B | XP_974632.1 | 17.6 | 14.8 | 15.4 | 19.9 | **27.4** | 19.3 | 20.7 | 19.3 |
| *Tc* | Group 4B | XP_974652.1 | 17 | 19 | 14.8 | 26.5 | **30.4** | 27.4 | 22.8 | 21.7 |
| *Zn* | Group 4A | XP_021927739.1^2^ | 19.9 | 21.1 | 16.2 | 25.2 | **30.2** | 18.6 | 20 | 17.4 |

^1^Sequence identity (as a percent) between the homologous region of each insect cytb561 and each *D. melanogaster* cytb561. The highest identity in each row is cell shaded and listed in bold. Group 4A sequences are shaded in pale orange and all other sequences are shaded in pale blue.

^2^Group 4A insect sequences with higher identity to a human cytb561 than a *D. melanogaster* cytb561.

^3^Edited to correct a gene prediction error.
