# Supplementary material for: Comparison of insect and human cytochrome b561 proteins: Insights into candidate ferric reductases in insects
Source: PLoS One. 2023 Dec 1;18(12):e0291564. doi: 10.1371/journal.pone.0291564 (PMC10691727; doi:10.1371/journal.pone.0291564)
Supplement: S3 Table — (DOCX) [file pone.0291564.s008.docx]

**S3 Table. ChimeraX-predicted contacts for Dcytb, CG1275, and Nemy with heme and ascorbate molecules.**

| **Ligand^1^** | **Position^2^** | **Dcytb Amino Acid** | **# Contacts^3^** | **# H-bonds^3^** | **Nemy Amino Acid** | **# Contacts** | **# H-bonds** | **CG1275 Amino Acid** | **# Contacts** | **# H-Bonds** | **Jalview Score^4^** |
| --- | --- | --- | --- | --- | --- | --- | --- | --- | --- | --- | --- |
| Heme (NC) | H1-2 | Trp30 | 3 |  | Trp79 | 1 |  | Trp126 | 1 |  | * |
| Heme (NC) | H1-3 | Phe47 | 5 |  | Phe97 | 10 |  | Phe144 | 12 |  | * |
| Ascorbate (NC) | H1-3 | Phe47 | 2 |  | Phe97 | 3 |  | Phe144 | 2 |  | * |
| Heme (NC) | H1 | His50 | 23 | 2 | His100 | 19 | 3 | His147 | 20 | 2 | * |
| Heme (NC) | H1+1 | Pro51 | 4 |  | Pro101 | 4 |  | Pro 148 | 6 |  | * |
| Heme (NC) | H1+4 | Met54 | 8 |  | Met104 | 7 |  | Met 151 | 10 |  | * |
| Heme (NC) | H1+8 | Phe58 | 1 |  | Phe108 | 3 |  | Phe155 | 3 |  | 1 |
| Heme (C) | H1+12 | Gln62 | 0 |  | Ser112 | 0 |  | Tyr159 | 4 |  | 4 |
| Heme (C) | H1+16 | Ile66 | 3 |  | Ile116 | 2 |  | Ile163 | 5 |  | 8 |
| Heme (C) | H1+19 | Tyr69 | 14 |  | Tyr119 | 9 |  | Tyr166 | 7 |  | * |
| Heme (C) | H1+20 | Arg70 | 5 | 1 | Arg120 | 0 |  | Arg167 | 4 | 1 | 9 |
| Heme (C) | H2-7 | Lys79 | 6 |  | Gln129 | 0 |  | Lys174 | 0 |  | 3 |
| Ascorbate (C) | H2-7 | Lys 79 | 6 | 1 | Gln129 | 0 |  | Lys174 | 4 | 1 | 3 |
| Heme (C) | H2-3 | Lys83 | 4 |  | Lys131 | 3 |  | Lys178 | 1 |  | * |
| Ascorbate (C) | H2-3 | Lys 83 | 4 | 2 | Lys131 | 6 | 2 | Lys178 | 4 | 2 | * |
| Heme (C) | H2 | His86 | 23 | 1 | His134 | 15 | 1 | His181 | 21 | 2 | * |
| Heme (C) | H2+1 | Ala87 | 2 |  | Met135 | 1 |  | Ala182 | 1 |  | 5 |
| Heme (C) | H2+4 | Asn90 | 7 |  | His138 | 12 |  | His185 | 17 |  | 4 |
| Heme (NC) | H2+15 | Val101 | 1 |  | Phe149 | 2 |  | Leu 196 | 3 |  | 6 |
| Heme (NC) | H2+18 | Val104 | 5 |  | Val152 | 8 |  | Val199 | 10 |  | 8 |
| Heme (NC) | H2+19 | Phe105 | 2 |  | Phe153 | 8 |  | Phe200 | 6 |  | 7 |
| Heme (NC) | H2+22 | His108 | 1 |  | His156 | 8 |  | His203 | 7 |  | * |
| Ascorbate (NC) | H2+22 | His108 | 2 | 1 | His156 | 2 | 1 | His203 | 1 | 1 | * |
| Ascorbate (NC) | H3-7 | Ile113 | 0 |  | Lys161 | 23 | 1 | Ile210 | 0 |  | 6 |
| Heme (NC) | H3-5 | Asn115 | 4 |  | Asn163 | 5 |  | Asn212 | 5 |  | 5 |
| Heme (NC) | H3-4 | Met116 | 1 | 1 | Phe164 | 0 | 1 | Met213 | 1 | 1 | 8 |
| Heme (NC) | H3-3 | Tyr117 | 1 |  | Tyr165 | 0 |  | Tyr214 | 0 |  | * |
| Heme (NC) | H3-2 | Ser118 | 4 | 2 | Ser166 | 4 | 1 | Ser215 | 4 | 1 | 8 |
| Heme (NC) | H3 | His120 | 16 |  | His168 | 30 |  | His217 | 24 |  | * |
| Heme (NC) | H3+1 | Ser121 | 3 |  | Ser169 | 9 |  | Ser218 | 1 |  | * |
| Heme (NC) | H3+4 | Gly124 | 4 |  | Gly172 | 6 |  | Gly221 | 4 |  | * |
| Heme (NC) | H3+5 | Leu125 | 0 |  | Phe173 | 1 |  | Leu222 | 1 |  | 7 |
| Heme (NC) | H3+7 | Ala127 | 0 |  | Thr175 | 1 |  | Ala224 | 0 |  | 7 |
| Heme (NC) | H3+8 | Val128 | 1 |  | Met176 | 0 |  | Val225 | 1 |  | 8 |
| Heme (C) | H3+11 | Tyr131 | 8 |  | Phe179 | 3 |  | Phe228 | 6 |  | 9 |
| Heme (C) | H3+14 | Gln134 | 6 |  | Gln182 | 2 |  | Gln231 | 0 |  | * |
| Heme (C) | H3+15 | Leu135 | 1 |  | Phe183 | 4 |  | Tyr232 | 4 |  | 7 |
| Heme (C) | H3+18 | Gly138 | 4 |  | Gly186 | 10 |  | Gly235 | 5 |  | * |
| Heme (C) | H3+19 | Phe139 | 3 |  | Phe187 | 4 |  | Phe236 | 4 |  | 6 |
| Heme (C) | H3+22 | Phe142 | 5 |  | Phe190 | 13 |  | Phe239 | 7 |  | 9 |
| Ascorbate (C) | H3+22 | Phe142 | 4 |  | Phe190 | 6 |  | Phe239 | 5 |  | 9 |
| Heme (C) | H3+23 | Leu143 | 5 |  | Leu191 | 0 |  | Leu240 | 4 |  | 6 |
| Ascorbate (C) | H4-7 | Arg152 | 5 | 1 | Arg204 | 10 | 3 | Arg249 | 6 |  | 9 |
| Ascorbate (C) | H4-6 | Ala153 | 0 |  | Ser205 | 9 | 1 | Ile250 | 4 |  | 3 |
| Heme (C) | H4-3 | Met156 | 3 |  | Val208 | 0 |  | Met253 | 3 |  | 5 |
| Ascorbate (C) | H4-3 | Met156 | 1 |  | Val208 | 6 |  | Met253 | 8 |  | 5 |
| Heme (C) | H4 | His159 | 14 |  | His211 | 31 | 2 | His256 | 20 | 1 | * |
| Heme (C) | H4+1 | Val160 | 2 |  | Ala212 | 4 |  | Ile257 | 2 |  | 5 |
| Heme (C) | H4+4 | Gly163 | 1 |  | Gly215 | 2 |  | Gly260 | 3 |  | * |
| Heme (C) | H4+5 | Ile164 | 1 |  | Leu216 | 0 |  | Leu261 | 2 |  | 9 |
| Heme (NC) | H4+11 | Val170 | 2 |  | Ala222 | 1 |  | Ala267 | 1 |  | 8 |
| Heme (NC) | H4+14 | Thr173 | 4 |  | Thr225 | 12 |  | Ser270 | 2 |  | 7 |
| Heme (NC) | H4+15 | Ala174 | 3 |  | Ser226 | 7 |  | Ala271 | 3 |  | 7 |
| Heme (NC) | H4+18 | Gly177 | 5 |  | Gly229 | 5 |  | Gly274 | 11 |  | * |
| Heme (NC) | H4+19 | Leu178 | 2 |  | Leu230 | 1 |  | Ile275 | 6 |  | 8 |
| Heme (NC) | H4+21 | Glu180 | 7 |  | Glu232 | 9 |  | Glu277 | 7 |  | 8 |
| Heme (NC) | H4+22 | Lys181 | 2 |  | Lys233 | 2 |  | Lys278 | 2 |  | 9 |
| Ascorbate (NC) | H4+25 | Phe184 | 4 |  | Glu236 | 29 |  | Phe281 | 2 |  | 4 |
| Heme (C) | n/a | Val219 | 2 |  | Val273 | 1 |  | Ala316 | 0 |  | 7 |
| Heme (C) | n/a | Lys225 | 5 | 2 | Pro279 | 0 |  | Lys332 | 0 |  | 3 |

^1^Ligand: (C) = cytoplasmic; (NC) = non-cytoplasmic.

^2^Position: Relative to H1-H4 (heme-coordinating histidines).

^3^Number of contacts and H-bonds separately predicted using ChimeraX.

^4^Jalview Scores are based on the Figure 4 alignment. Higher scores correspond to higher conservation with an * for entirely conserved.
