# Supplementary material for: Comparison of insect and human cytochrome b561 proteins: Insights into candidate ferric reductases in insects
Source: PLoS One. 2023 Dec 1;18(12):e0291564. doi: 10.1371/journal.pone.0291564 (PMC10691727; doi:10.1371/journal.pone.0291564)
Supplement: S4 Table — (DOCX) [file pone.0291564.s009.docx]

**S4 Table. AlphaFold model confidence for *D. melanogaster* CG1275, Nemy, and CG8399 structures based on per-residue confidence score (pLDDT) between 0 and 100.**

| **Region of**  **CG1275 Isoform A** | **% Very Low (pLDDT <50)** | **% Low (70 > pLDDT > 50)** | **% Confident**  **(90 > pLDDT > 70)** | **% Very High (pLDDT > 90)** |
| --- | --- | --- | --- | --- |
| N-terminus  Amino acids 1-99 | 93.9% (93/99) | 5.1% (5/99) | 1.0% (1/99) | 0% (0/99) |
| Full cytb561 domain  Amino acids 100-317 | 0% (0/218) | 0% (0/218) | 2.3% (5/218) | 97.7% (213/218) |
| C-terminus  Amino acids 318-340 | 4.4% (1/23) | 39.1% (9/23) | 30.4% (7/23) | 26.1% (6/23) |

| **Region of**  **Nemy Isoform A** | **% Very Low (pLDDT <50)** | **% Low**  **(70 > pLDDT > 50)** | **% Confident**  **(90 > pLDDT > 70)** | **% Very High (pLDDT > 90)** |
| --- | --- | --- | --- | --- |
| N-terminus  Amino acids 1-53 | 75.5% (40/53) | 18.9% (10/53) | 5.7% (3/53) | 0% (0/53) |
| Full cytb561 domain  Amino acids 54-274 | 0% (0/221) | 0% (0/221) | 17.2% (38/221) | 82.8% (183/221) |
| C-terminus  Amino acids 275-290 | 87.5% (14/16) | 6.25% (1/16) | 6.25% (1/16) | 0% (0/16) |

| **Region of**  **CG8399 Isoform A** | **% Very Low (pLDDT <50)** | **% Low**  **(70 > pLDDT > 50)** | **% Confident**  **(90 > pLDDT > 70)** | **% Very High (pLDDT > 90)** |
| --- | --- | --- | --- | --- |
| Signal peptide  Amino acids 1-30 | 76.7% (23/30) | 23.3% (7/30) | 0% (0/30) | 0% (0/30) |
| Reeler domain  Amino acids 31-184 | 0% (0/154) | 2.6% (4/154) | 40.3% (62/154) | 57.1% (88/154) |
| Linker (between reeler and DOMON domains)  Amino acids 185-223 | 87.2% (34/39) | 10.3% (4/39) | 2.6% (1/39) | 0% (0/39) |
| DOMON domain  Amino acids 224-400 | 0% (0/177) | 0% (0/177) | 37.9% (67/177) | 62.1% (110/177) |
| Linker (between DOMON & cytb561 domains)  Amino acids 401-403 | 0% (0/3) | 0% (0/3) | 100% (3/3) | 0% (0/3) |
| Full cytb561 domain  Amino acids 404-636 | 0% (0/233) | 9.87% (23/233) | 21.46% (50/233) | 68.67% (160/233) |
| Cytb561 core domain^1^ (helices 1-4 and their loops)  Amino acids 404-535 | 0% (0/132) | 0% (0/132) | 18.2% (24/132) | 81.8% (108/132) |
| C-terminus  Amino acids 637-647 | 27.3% (3/11) | 36.4% (4/11) | 36.4% (4/11) | 0% (0/11) |

^1^A subset of the residues in the row above to emphasize that the core domain prediction is much better than the full cytb561 domain prediction.
