# Supplementary material for: Comparison of insect and human cytochrome b561 proteins: Insights into candidate ferric reductases in insects
Source: PLoS One. 2023 Dec 1;18(12):e0291564. doi: 10.1371/journal.pone.0291564 (PMC10691727; doi:10.1371/journal.pone.0291564)
Supplement: S5 Table — (DOCX) [file pone.0291564.s010.docx]

**S5 Table.** **Insect sequences with similar extended regions to *A. pisum* Nemy.**

| **Order**  ***Species* (common name)** | **Accession number^1^** |
| --- | --- |
| Hemiptera  *Aphis craccivora* (cowpea aphid) | KAF0754846.1 |
| Hemiptera  *Aphis glycines* (soybean aphid) | KAE9533855.1 |
| Hemiptera  *Aphis gossypii* (cotton aphid) | XP_027844671.1 |
| Hemiptera  *Cinara cedri* (cedar bark aphid) | VVC36571.1 |
| Hemiptera  *Diuraphis noxia* (Russian wheat aphid) | XP_015366894.1 |
| Hemiptera  *Melanaphis sacchari* (sugarcane aphid) | XP_025196103.1 |
| Hemiptera  *Myzus persicae* (green peach aphid) | XP_022171233.1 |
| Hemiptera  *Rhopalosiphum maidis* (corn aphid) | XP_026816157.1 |
| Hemiptera  *Sipha flava* (yellow sugarcane aphid) | XP_025407957.1 |

^1^Sequences identified from a BLAST search using query “XP_00194292276.1,” the *A. pisum* Nemy sequence with approximately 150 additional N-terminal amino acids and an extended non-cytoplasmic loop connecting helix 1 and helix 2, against the class Insecta.
