# Supplementary material for: Comparison of insect and human cytochrome b561 proteins: Insights into candidate ferric reductases in insects
Source: PLoS One. 2023 Dec 1;18(12):e0291564. doi: 10.1371/journal.pone.0291564 (PMC10691727; doi:10.1371/journal.pone.0291564)
Supplement: S6 Table — (DOCX) [file pone.0291564.s011.docx]

**S6 Table. Insect sequences with two DOMON domains.**

| **Order**  ***Species* (common name)** | **Accession number^1^** |
| --- | --- |
| Hemiptera  *Aphis craccivora* (cowpea aphid) | KAF0772237.1 |
| Hemiptera  *Aphis glycines* (soybean aphid) | KAE9523894.1 |
| Hemiptera  *Aphis gossypii* (melon aphid) | XP_027841628.1 |
| Hemiptera  *Cinara cedri* (cedar bark aphid) | VVC24734.1 |
| Hemiptera  *Diuraphis noxia* (Russian wheat aphid) | XP_015375719.1 |
| Hemiptera  *Melanaphis sacchari* (sugarcane aphid) | XP_025198216.1 |
| Hemiptera  *Rhopalosiphum maidis* (corn aphid) | XP_026806269.1 |
| Hemiptera  *Sipha flava* (yellow sugarcane aphid) | XP_025420155.1 |
| Hemiptera  *Myzus persicae* (green peach aphid) | XP_022176482.1 |
| Hemiptera  *Apolygus lucorum* (small green plant bug) | KAF6216808.1 |
| Hemiptera  *Bemisia tabaci* (silverleaf whitefly) | XP_018904139.1 |
| Hemiptera  *Cimex lectularius* (bed bug) | XP_014245063.1 |
| Hemiptera  *Diaphorina citri* (Asian citrus psyllid) | XP_017300331.1 |
| Hemiptera  *Laodelphax striatellus* (small brown planthopper) | RZF35324.1 |
| Hemiptera  *Halyomorpha halys* (brown marmorated stink bug) | XP_014284211.1 |
| Hemiptera  *Nilaparvata lugens* (brown planthopper) | XP_039277083.1 |
| Hemiptera  *Homalodisca vitripennis* (glassy-winged sharpshooter) | KAG8312720.1 |
| Coleoptera  *Diabrotica vergifera* (corn rootworm) | XP_028141846.1 |
| Coleoptera  *Leptinotarsa decemlineata* (Colorado potato beetle) | XP_023022567.1 |

^1^Sequences identified from a BLAST search using query “XP_001950579.2,” the *A. pisum* CG8399 sequence with two DOMON domains, against the class Insecta.
