# Supplementary material for: Comparison of insect and human cytochrome b561 proteins: Insights into candidate ferric reductases in insects
Source: PLoS One. 2023 Dec 1;18(12):e0291564. doi: 10.1371/journal.pone.0291564 (PMC10691727; doi:10.1371/journal.pone.0291564)
Supplement: S7 Table — (DOCX) [file pone.0291564.s012.docx]

**S7 Table.** **Single-domain insect sequences with highest similarity to CG8399.**

| **Order**  ***Species* (common name)** | **Accession number^1^** |
| --- | --- |
| Diptera  *Aedes aegypti* (Yellow fever mosquito) | XP_021698251.1 |
| Diptera  *Aedes albopictus* (Asian tiger mosquito) | XP_029708302.1 |
| Diptera  *Anopheles albimanus* (New world malaria mosquito) | XP_035793296.1 |
| Diptera  *Anopheles arabiensis* (African malaria mosquito) | XP_040158133.1 |
| Diptera  *Anopheles coluzzii* (African malaria mosquito) | XP_040226982.1 |
| Diptera  *Anopheles darlingi* (American malaria mosquito) | ETN60734.1 |
| Diptera  *Anopheles merus* (mosquito) | XP_041769842.1 |
| Diptera  *Anopheles sinensis* (mosquito) | KFB44363.1 |
| Diptera  *Anopheles stephensi* (Indo-Pakistan malaria mosquito) | XP_035914634.1 |
| Diptera  *Culex quinquefasciatus* (southern house mosquito) | XP_038118957.1 |
| Diptera  *Bradysia coprophila* (darkwinged fungus gnat) | XP_037036556.1 |
| Diptera  *Bradysia odoriphaga* (darkwinged fungus gnat) | KAG4076368.1 |

^1^Sequences identified from a BLAST search using query “XP_314065.2,” the *A. gambiae* CG8399 single-domain cytb561 sequence lacking both reeler and DOMON domains, against the class Insecta.
