# Supplementary material for: Comparison of insect and human cytochrome b561 proteins: Insights into candidate ferric reductases in insects
Source: PLoS One. 2023 Dec 1;18(12):e0291564. doi: 10.1371/journal.pone.0291564 (PMC10691727; doi:10.1371/journal.pone.0291564)
Supplement: S8 Table — (DOCX) [file pone.0291564.s013.docx]

**S8 Table. Predicted subcellular localization of insect cytb561 sequences based on DEEPLOC-1.0 webserver.**

| **Accession number** | **Group** | **Predicted Localization** | **Cell membrane^1^** | **ER^2^** | **Golgi** | **Lysosome** |
| --- | --- | --- | --- | --- | --- | --- |
| XP_015837014.1_Tc | Group 4 | ER | 0.1201 | 0.3888 | 0.2281 | 0.2626 |
| XP_974632.1_Tc | Group 4 | Golgi | 0.0647 | 0.2818 | 0.3967 | 0.2562 |
| XP_974652.1_Tc | Group 4 | ER | 0.0748 | 0.4115 | 0.2891 | 0.2244 |
| XP_008195477.1_Tc | Group 4 | Golgi | 0.0352 | 0.2676 | 0.5114 | 0.1855 |
| XP_552919.3_Ag | Group 4 | Golgi | 0.1246 | 0.202 | 0.3427 | 0.3298 |
| XP_001238089.2_Ag | Group 4 | Golgi | 0.1175 | 0.2737 | 0.3197 | 0.2855 |
| XP_013172802.1_Px | Group 4 | Lysosome | 0.0786 | 0.1172 | 0.2758 | 0.5216 |
| XP_013162686.1_Px | Group 4 | Lysosome | 0.1012 | 0.1563 | 0.3461 | 0.3942 |
| XP_013172799.1_Px | Group 4 | Golgi | 0.1573 | 0.1845 | 0.3784 | 0.2769 |
| XP_013172813.1_Px | Group 4 | Golgi | 0.106 | 0.2345 | 0.3583 | 0.2996 |
| XP_013162691.1_Px | Group 4 | Lysosome | 0.0933 | 0.3079 | 0.2671 | 0.3288 |
| XP_013172804.1_Px | Group 4 | Golgi | 0.0955 | 0.3178 | 0.3287 | 0.2489 |
| NP_609982.1_Dm | Group 4 | Golgi | 0.1152 | 0.2663 | 0.3303 | 0.2876 |
| NP_609986.1_Dm | Group 4 | Lysosome | 0.0881 | 0.2983 | 0.2308 | 0.3821 |
| NP_570039.1_Dm | Group 4 | Lysosome | 0.1743 | 0.1386 | 0.2253 | 0.4613 |
| XP_003249671.1_Am | Group 4 | Golgi | 0.143 | 0.1862 | 0.3732 | 0.2959 |
| NP_001260592.1_Dm | Group 4 | Golgi | 0.0811 | 0.1634 | 0.4081 | 0.3457 |
| NP_609989.1_Dm | Group 4 | Golgi | 0.0549 | 0.2851 | 0.3584 | 0.3006 |
| XP_001122176.1_Am | Group 4A | Lysosome | 0.1158 | 0.191 | 0.3412 | 0.3515 |
| XP_026469480.1_Cf | Group 4A | Lysosome | 0.0894 | 0.2834 | 0.2528 | 0.3723 |
| XP_026469242.1_Cf | Group 4A | Lysosome | 0.1185 | 0.1749 | 0.2805 | 0.423 |
| XP_008201603.1_Tc | Group 4A | ER | 0.099 | 0.3092 | 0.297 | 0.2901 |
| XP_320673.4_Ag | Group 4A | Golgi | 0.0653 | 0.2733 | 0.3963 | 0.2633 |
| XP_013174961.1_Px | Group 4A | Lysosome | 0.1475 | 0.171 | 0.2854 | 0.3939 |
| XP_021927739.1_Zn | Group 4A | Lysosome | 0.1565 | 0.1701 | 0.2632 | 0.4072 |
| XP_314065.2_Ag | CG8399 | Lysosome | 0.3707 | 0.04 | 0.0956 | 0.4929 |
| XP_001950579.2_Ap | CG8399 | Cell membrane | 0.8756 | 0.0243 | 0.0083 | 0.0854 |
| XP_396579.3_Am | CG8399 | Cell membrane | 0.8254 | 0.0605 | 0.0097 | 0.0967 |
| NP_611079.2_Dm | CG8399 | Cell membrane | 0.8186 | 0.0515 | 0.0132 | 0.1102 |
| XP_013164083.1_Px | CG8399 | Cell membrane | 0.7493 | 0.0731 | 0.0383 | 0.125 |
| XP_002423127.1_Ph | CG8399 | Cell membrane | 0.7929 | 0.0928 | 0.0272 | 0.0807 |
| XP_021919699.1_Zn | CG8399 | Cell membrane | 0.8736 | 0.0068 | 0.0093 | 0.1091 |
| XP_015836986.1_Tc | CG8399 | Cell membrane | 0.7084 | 0.1181 | 0.0248 | 0.1427 |
| XP_314066.4_Ag | CG8399 | Cell membrane | 0.7869 | 0.1533 | 0.0153 | 0.0371 |
| XP_026481553.1_Cf | CG8399 | Cell membrane | 0.7945 | 0.1173 | 0.0141 | 0.0683 |
| XP_021939496.1_Zn | Nemy | Lysosome | 0.2449 | 0.1184 | 0.2518 | 0.3468 |
| XP_008198104.1_Tc | Nemy | Lysosome | 0.2305 | 0.1041 | 0.2183 | 0.4366 |
| XP_026473332.1_Cf | Nemy | Lysosome | 0.2066 | 0.1602 | 0.2203 | 0.4121 |
| XP_002430226.1_Ph | Nemy | Lysosome | 0.2073 | 0.0679 | 0.1966 | 0.52 |
| NP_001298968.1_Px | Nemy | Lysosome | 0.2463 | 0.1244 | 0.1888 | 0.4396 |
| NP_001163128.1_Dm | Nemy | Lysosome | 0.1882 | 0.1741 | 0.264 | 0.373 |
| XP_314126.2_Ag | Nemy | Lysosome | 0.1955 | 0.1699 | 0.2699 | 0.3641 |
| XP_001949276.1_Ap | Nemy | Lysosome | 0.3695 | 0.0729 | 0.0951 | 0.4533 |
| NP_001155374.1_Ap | CG1275 | Lysosome | 0.1501 | 0.0912 | 0.2134 | 0.5447 |
| XP_001950854.1_Ap | CG1275 | Lysosome | 0.1826 | 0.1045 | 0.2396 | 0.4725 |
| NP_001280323.1_Ap | CG1275 | Lysosome | 0.2415 | 0.0923 | 0.1549 | 0.5108 |
| XP_003246890.1_Ap | CG1275 | Lysosome | 0.2196 | 0.0728 | 0.2247 | 0.4798 |
| XP_008194670.1_Tc | CG1275 | Lysosome | 0.2689 | 0.0246 | 0.1127 | 0.5917 |
| XP_006572086.1_Am | CG1275 | Lysosome | 0.1834 | 0.0678 | 0.1523 | 0.5951 |
| XP_026462102.1_Cf | CG1275 | Lysosome | 0.1678 | 0.0711 | 0.1694 | 0.5907 |
| XP_021935166.1_Zn | CG1275 | Lysosome | 0.1792 | 0.1226 | 0.1842 | 0.5133 |
| XP_002426701.1_Ph | CG1275 | Lysosome | 0.3033 | 0.093 | 0.1123 | 0.4891 |
| XP_315519.4_Ag | CG1275 | Lysosome | 0.3231 | 0.0566 | 0.1535 | 0.4559 |
| NP_995963.1_Dm | CG1275 | Lysosome | 0.3271 | 0.0744 | 0.0792 | 0.5166 |

^1^The color coding was done in Excel using the Green-Yellow-Red color scale (with green for the highest value, yellow for the midpoint/50th percentile value, and red for the lowest value) across the whole data set (all four columns).

^2^ER is endoplasmic reticulum.
